# Cellular Plasticity Enables Adaptation to Unforeseen Cell-Cycle Rewiring Challenges

**DOI:** 10.1371/journal.pone.0045184

**Published:** 2012-09-18

**Authors:** Yair Katzir, Elad Stolovicki, Shay Stern, Erez Braun

**Affiliations:** 1 Faculty of Medicine, Technion, Haifa, Israel; 2 Department of Physics, Technion, Haifa, Israel; 3 Laboratory of Network Biology, Technion, Haifa, Israel; 4 Department of Biological Chemistry, Weizmann Institute of Science, Rehovot, Israel; CRG, Spain

## Abstract

The fundamental dynamics of the cell cycle, underlying cell growth and reproduction, were previously found to be robust under a wide range of environmental and internal perturbations. This property was commonly attributed to its network structure, which enables the coordinated interactions among hundreds of proteins. Despite significant advances in deciphering the components and autonomous interactions of this network, understanding the interfaces of the cell cycle with other major cellular processes is still lacking. To gain insight into these interfaces, we used the process of genome-rewiring in yeast by placing an essential metabolic gene *HIS3* from the histidine biosynthesis pathway, under the exclusive regulation of different cell-cycle promoters. In a medium lacking histidine and under partial inhibition of the HIS3p, the rewired cells encountered an unforeseen multitasking challenge; the cell-cycle regulatory genes were required to regulate the essential histidine-pathway gene in concert with the other metabolic demands, while simultaneously driving the cell cycle through its proper temporal phases. We show here that chemostat cell populations with rewired cell-cycle promoters adapted within a short time to accommodate the inhibition of HIS3p and stabilized a new phenotypic state. Furthermore, a significant fraction of the population was able to adapt and grow into mature colonies on plates under such inhibiting conditions. The adapted state was shown to be stably inherited across generations. These adaptation dynamics were accompanied by a non-specific and irreproducible genome-wide transcriptional response. Adaptation of the cell-cycle attests to its multitasking capabilities and flexible interface with cellular metabolic processes and requirements. Similar adaptation features were found in our previous work when rewiring *HIS3* to the GAL system and switching cells from galactose to glucose. Thus, at the basis of cellular plasticity is the emergence of a yet-unknown general, non-specific mechanism allowing fast inherited adaptation to unforeseen challenges.

## Introduction

The living cell is a dynamical system demonstrating considerable organization manifested in its metabolism, morphology and function. Cell cycle regulation, which is responsible for proper cell growth and division, coordinates a temporal phenotypic order that enables this dynamic behavior. Understanding the internal regulation of the cell cycle as well as its interface with numerous other cellular processes is therefore fundamental to many fields of biological research, such as development and cancer. The operational principles of the eukaryotic cell cycle have been found to be universal across a wide range of organisms, from yeast to mammals [1], [2], [3], [4]. The common picture emerging is of the cell cycle progression driven by a robust machinery, presumably an outcome of a scrutinized evolutionary natural selection process [5], [6], [7], [8], [9]. However, direct experimental evidences for the mechanisms underlying this robustness are still lacking. Thus, despite the success in deciphering the cell cycle circuitry and genomic makeup, two basic inter-related issues beyond its autonomous normal operation remain largely open: its flexibility to respond to environmental stresses and accommodate internal perturbations [10], and its interface with the other intracellular processes, in particular the metabolic system [11], [12], [13].

The cell-cycle progression is regulated at two levels: via protein-protein interactions, the main components of which are cyclins (which bind to cyclin-dependent kinases (CDKs)) and their degraders (e.g. anaphase promoting complex (APC)), and via protein-DNA interactions (transcription factors (TFs)) [14]. Recent studies have revealed that the major transitions between phases in the cycle are transcriptionally regulated [15], but there are also important check-points involving other mechanisms at various phases of the cycle [16] along with an essential feedback mechanism to ensure coherent entry into the cycle [17]. Evidently, the cell-cycle network is not an autonomous isolated “oscillator”, but rather an integral part of the cellular complex web of interactions. Indeed, it has been demonstrated that in the budding yeast hundreds of genes (∼800) that do not directly participate in the cell cycle process exhibit temporal dynamics similar to the genes that directly regulate the cell cycle progression, and are synchronized with its dynamic phases [12], [18]. However, the functional significance of this temporal ordering remains elusive.

In this paper we use a genome-rewiring methodology to open a window to these dynamical aspects of the cell cycle, in particular the flexibility of its interface with the metabolic system. We focus here on transcriptional regulation and introduce a direct regulatory perturbation by rewiring the genome; placing a foreign, essential metabolic gene exclusively under one of the promoters of the cell cycle in the budding yeast [19]. Such genome rewiring events are not completely artificial, as they are thought to play an important role in the emergence of novel phenotypes in the evolution of developmental systems [20], [21], [22], [23]. We have recently shown in a separated experimental system, that rewiring an essential metabolic gene to a foreign regulatory system and creating an unforeseen challenge, is an effective way for perturbing the cellular regulatory modes. The genome rewiring perturbation exposes novel adaptive responses that are based on non-specific cellular plasticity mechanisms [19], [24], [25]. Our approach of genome rewiring has several advantages over the more commonly used methodologies that rely on gene deletion, mutations in coding regions or common environmental stresses. First, it does not introduce new proteins or other intracellular components, nor deletes existing ones. Rather, it re-shuffles existing cellular interactions, forcing proteins to operate in novel contexts. Second, it allows to study the response of cells to unforeseen challenges not previously encountered in their evolutionary history. This is significant since it has the potential to expose new cellular dynamics and mechanisms that otherwise might be masked by the operation of specific “hard-wired” functional modules that were selected in evolution to respond in a direct way to familiar stresses. In the case of the cell cycle, its promoters are “hard-wired” to perform the routine task of cell cycle progression but their potential “soft” interactions with other cellular processes and regulators remain hidden until exposed by novel perturbations. Harnessing cell-cycle regulators to directly control an essential metabolic process increases the load on its regulatory network at a specific phase of the cycle and demands re-distribution of its resources. Such a perturbation introduces a complex challenge to the cells by requiring the cell-cycle regulators to operate outside of their natural context and in concert with arbitrarily chosen metabolic demands.

We show here that yeast cell populations with rewired cell-cycle promoters can rapidly adapt to grow at normal rates despite an increased inhibition of the metabolic rewired gene. Moreover, the new adapted phenotype is stably inherited across generations. A significant fraction of the population has the potential to adapt to the severe unforeseen genome-rewiring challenge. Thus, the ability to adapt is not a special property of a rare subpopulation. Furthermore, we show that underlying the adaptation process there is a non-specific and irreproducible genome-wide transcriptional response. The cell cycle system is regarded as a tightly regulated network, ensuring its robust temporal ordered dynamics. Our current experiments do not address a specific mechanism for adaptation, but they show that the cell cycle system forms a flexible and adaptive interface with the cellular metabolic processes. This interface can support multitasking utility of the cell-cycle promoters and enables concurrent regulation of their native function and the control of a foreign essential metabolic gene under inhibiting conditions. This adaptation, stimulated initially by a local perturbation to the cell-cycle network, is eventually manifested in a global re-organization of the cell’s regulatory modes and metabolic fluxes. As discussed below, these results prove the existence of a general and non-specific cellular mechanism allowing adaptation to unforeseen challenges. Thus, even the cell-cycle with its unique role, specific structure and tight regulation is able to interface flexibly with the other cellular processes. It suggests that robustness of temporal phenotypic order is not an autonomous property of the cell-cycle network, but rather is a property of the cellular system - the plasticity of the cellular integrated web of interactions.

## Results

**Figure 1:**
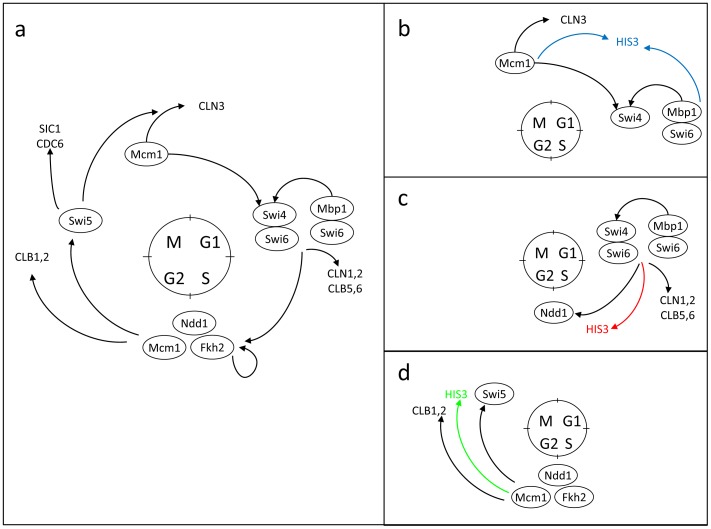
Rewiring the cell cycle network. (a) A schematic diagram of the cell cycle transcription regulation (based on ref [15] with modifications). Rewiring *HIS3* to be exclusively regulated by a duplicate of the cell cycle promoter regulating: (b) *Swi4*, (c) *Ndd1*, and (d) *Swi5*. Note that the native genes of the cell cycle network and their regulatory regions remained intact.

Experiments were performed on the haploid budding yeast *Saccharomyces cerevisiae*. The essential gene *HIS3,* from the histidine biosynthesis pathway, was detached from its native regulatory system and integrated into the genome under a particular promoter of the cell cycle. We have shown before in detailed experiments that *HIS3* is not redundant and does not have an alternative pathway in the budding yeast [19]. Moreover, under genome-rewiring conditions in which *HIS3* is rewired to be under the exclusive control of a foreign regulatory promoter (pGAL1 in our previous work), the cells could not produce an alternative metabolic pathway or detour the challenge by other regulatory pathways that do not involve the regulation of the rewired *HIS3* [19], [24], [25]. Thus, it is expected that also in the case of the cell-cycle promoters, cells will need to adapt the *HIS3* expression without detouring the regulatory challenge. We constructed a set of strains, each with *HIS3* rewired to be under the exclusive control of a different single cell-cycle’s promoter, regulating the genes *SWI4*, *NDD1* or *SWI5* (Fig. 1). Rewiring was carried out by duplicating one of the native cell-cycle’s regulating promoter, leaving the natural promoter intact, and integrating the *HIS3*’s ORF under this promoter into the genome (see *Methods*). These promoters were arbitrarily chosen, but they represent a set of essential transcription factors; each of them is known to activate a set of genes at different phases of the cycle. *SWI4* was shown to be part of the SBF regulatory complex activated during late G1 phase of the cycle to induce the expression of *NDD1,* which itself participates in another gene complex activating the G2 to M phase progression [15]. This latter complex activates *SWI5* which in turn is part of the process activating the genes responsible for the M to G1 phase progression, thus closing the cycle (Fig. 1a). By rewiring the promoter of each of these genes to regulate exclusively the essential metabolic *HIS3* gene, in parallel of its native regulation of the cell cycle process (Figs. 1b-d), we were able to compare the susceptibility of these essential nodes of the cell cycle network to the rewiring perturbation. The rewired promoters are restricted to operate by the cell-cycle’s limiting resources and only at short time intervals during a specific phase of the cycle. These constraints supposedly limit their ability to support the demands imposed by the histidine pathway and other metabolic processes, enabling us to present the cells with severe unforeseen challenges.

In a batch culture with medium lacking histidine, all the rewired strains (with *HIS3* linked to any one of the above-mentioned cell cycle promoters) could grow with no apparent significant effect on their growth rate or morphology compared to that in a medium containing histidine (Table 1). To challenge the cells, we utilized *3-amino-1,2,4-triazole* (*3AT)*, a specific competitive inhibitor of HIS3p, to increase the load on the cell populations in a controlled manner. *3AT* is a known drug, demonstrated to have minimal side effects on yeast beyond the specific inhibition of HIS3p [19], [25], [26], [27]. The growth rates of all the rewired strains in a medium containing 4 mM of *3AT* was much reduced compared to that in the same medium lacking *3AT* (Table 1). Similarly, the growth of colonies on agar plates with media lacking histidine but containing different concentrations of *3AT,* showed a significant delay in growth for all rewired cells; the first mature colonies appeared on the plates only after more than 4 days compared to the normal 2-days growth in a *3AT*-free medium (Fig. S1). It is clear that the presence of the HIS3p’s inhibiting drug in the medium caused a substantial challenge to the cells. As shown below, we indeed observed a direct stress due to effects of the challenge on the cell cycle, manifested by morphological changes during growth in the *3AT* environment. We have previously demonstrated that even a partial inhibition of the activity of HIS3p by *3AT* is a challenge to the rewired cells having *HIS3* under the exclusive regulation of a foreign promoter. Overcoming it requires a significant adaptation that involves the emergence of new regulatory modes [19], [24], [25].

**Table 1:** Growth exponents of the three strains in batch experiments.

histidine	+	−	−
**3AT(mM)**	0	0	4
**τ^−1^ (1/h) pSwi4**	0.240	0.363	0.088
**τ^−1^ (1/h) pNdd1**	0.201	0.202	0.018
**τ^−1^ (1/h) pSwi5**	0.231	0.236	0.079

All clones were grown over more than 80 hours with or without histidine in the medium and with different concentrations of *3AT*. The OD measurements of the growing batch cultures were fitted with exponential functions e^t/τ^.

**Figure 2:**
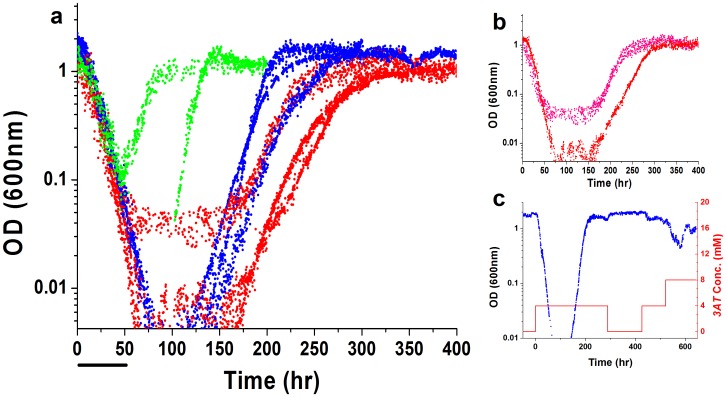
Population growth dynamics of rewired strains. (a) The cell density as measured by the optical density in repetitive chemostat experiments with the three rewired strains: blue, pSwi4-*HIS3* (Fig. 1b), 3 experiments; red, pNdd1-*His3* (Fig. 1c), 3 experiments, and green pSwi5-*HIS3* (Fig. 1d), 2 experiments. The chemostats were stabilized at steady state in a medium lacking *3AT* and were switched to the same medium supplemented with 4 mM *3AT* at t = 0. Note the logarithmic y-axis. Bar-10 chemostat generations. Note the variability between repeated experiments. (b) The chemostat growth dynamics of “twin” populations of the strain pNdd1-*HIS3*, derived from a single steady-state mother population and decoupled prior to the switch into the challenging *3AT* medium. Switching to a medium with 4 mM *3AT* was done at t = 0. These chemostats are the same as two of the chemostats (red) shown in (a). (c) **Inherited adaptation**. A time-extended chemostat experiment with the strain pSwi4-*HIS3* showing that after the establishment of an adapted state in 4 mM *3AT*, removing the *3AT* from the medium almost did not have any effect on the population growth dynamics, while switching to 8 mM *3AT* caused a slight decline in the population density followed by re-adaptation to the new medium. The red curve depicts the concentration of *3AT*.

**Figure 3:**
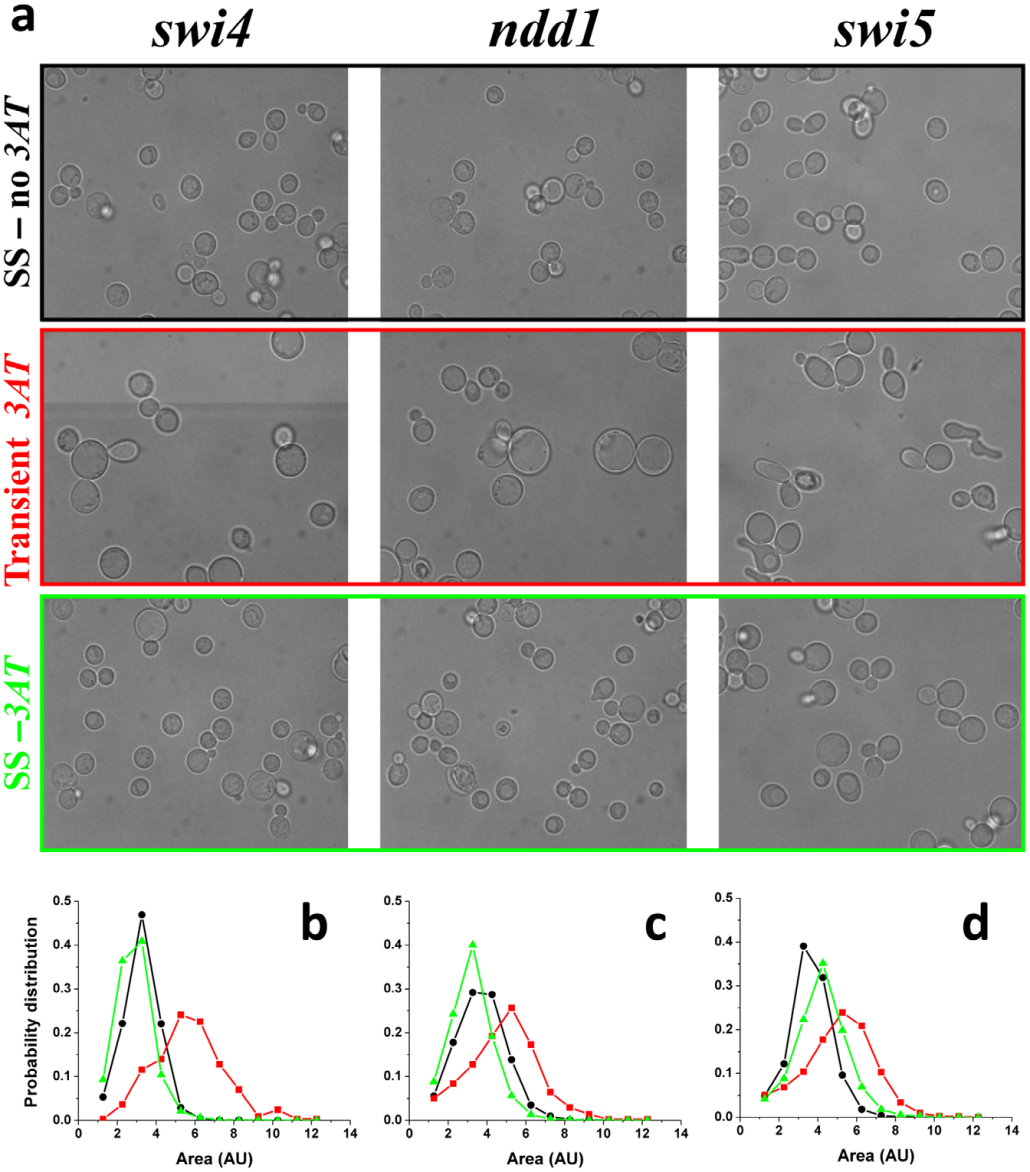
Morphology of adapting cells. (a) For each of the three strains of Figs. 1b–d, a sample of cells was harvested from the chemostat at three time points: initial steady-state with no *3AT* (upper row), during the declining phase in cell density after the addition of 4 mM of *3AT* (middle row), and at the adapted steady state (lower row). Histograms of cell size distributions for the three phases (black-initial; red-declining transient phase; green-adapted state) for: (b) pSwi4-*HIS3* (number of cells analyzed: initial-1102, transient-330, adapted-1340 ) (c) pNdd1-*HIS3* (number of cells analyzed: initial-1454, transient −794, adapted −1426) and (d) pSwi5-*HIS3* (number of cells analyzed: initial-3950, transient-3165, adapted-3559). The histograms were normalized to unit area and thus represent the probability density.

We next study the population growth dynamics in the challenging environment. Rewired cells were grown in a home-made chemostat; a continuous culture technique allowing growth of large cell populations over extended periods under constant environmental conditions [19], [28]. Our chemostat setup was constructed to enable the measurements of the population’s growth dynamics at high temporal resolution in conjunction with gene expression and cell morphology (*Methods*). Figure 2a depicts the results of repeated chemostat experiments, showing the growth dynamics of cell populations, as measured by optical density (OD), for strains with *HIS3* placed exclusively under the pSwi4, pNdd1 or pSwi5 promoters. The chemostat medium was free of histidine throughout the experiments. First, a steady state was established for all the populations in a *3AT*-free medium, followed by the switch at t = 0 to the same medium supplemented with 4 mM *3AT.* All populations exhibited a sharp exponential decline in cell density immediately upon exposure to the HIS3p inhibitor, proving again that the addition of the HIS3p inhibitor caused a significant challenge to the cells. Note that the decline at the onset of exposure to *3AT* was insensitive to the particular strain and was repeated with similar exponents in all experiments. The exponential decline in cell density at this transient phase (with mean exponential decay time ∼14±2 hr) was slower than the chemostat dilution time (∼7 hr), showing that there was a considerable cell growth during this period, albeit at a rate lower than that required to support steady growth under the chemostat dilution. This observation is also supported by direct imaging of the cells at this phase (see Fig. 3). We emphasize that the decline in cell density during this phase mainly reflects a population-average growth rate smaller than the chemostat’s dilution rate rather than cell arrest or cell death. Note also that the response for the application of *3AT* to the chemostat’s medium is instantaneous. The lack of delay in response suggests that there were no significant *HIS3*’s mRNA or HIS3p storages that played significant role at the onset of response to the inhibiting drug. The chemostat cell density reflected the integrated metabolic processes contributing to cell growth and proliferation and thus was sensitive to the overall metabolic state of the cells as well as to the functioning of the cell cycle [29]. The limited-resource environment and the dilution rate dictated by the chemostat, intensify the cell density as a sensitive measure of the metabolic and cell cycle response of the population. Thus, the decline of the cell density in the chemostat in response to the addition of *3AT*, reflected a stressful load on the cells’ growth capabilities.

Interestingly, all populations accommodated to the challenging environment within a variable time period spanning 10–40 chemostat-generation times, after which a stable steady state was established at approximately the same OD level as the one prior to the introduction of *3AT*. This indicates an inherent robustness of the cell-cycle regulatory system, allowing it to maintain homeostasis by reaching a similar metabolic state (manifested by the chemostat steady-state cell density) under different conditions – with and without the HIS3p inhibition. Note that this was not necessarily a direct response of the cell cycle system: rather it may reflect the interface of the cell cycle with the cell’s metabolic system. As shown below, the emergence of a new phenotypic state under *3AT*, represents true adaptation; it was stably inherited along generations. The p*Swi5*-*HIS3* strain (Fig. 2a green curves) exhibited shorter adaptation time compared with the p*Swi4*-*HIS3* (Fig. 2a blue curves) and p*Ndd1*-*HIS3* (Fig. 2a red curves) strains. Note, however, that the same strain may show variable response in repeated experiments, showing that there is a stochastic component in the adaptation process.

On agar plates a large number of individual cells, representing a sizeable fraction of the original population, adapted to grow into mature colonies on media lacking histidine and containing *3AT* (see Fig. S1 for a quantitative analysis). Within a certain *3AT*-concentration range (4–20 mM), the adaptation time scale depended on the amount of *3AT,* with clear differences between the different promoters. The variability in response of the different promoters proves that the adaptation process was not a mere metabolic response, but rather was sensitive to the regulatory constraints set by the specific rewiring event. Evidently, the presence of *3AT* caused a delay in adaptation that was proportional to the concentration of the inhibitor and consequently also a severe proportional reduction in the final number of adapted colonies above certain concentrations. Increasing amounts of *3AT* increased the inhibition of HIS3p and presumably drained significant resources from the cell cycle network to compensate the loss of active proteins by enhanced expression levels, presenting the cell populations with a growing challenge that scales with the concentration of the inhibitor. For a given number of plated cells, there was a clear upper limit to the concentration of *3AT*, depending on the specific rewired promoter, above which no observable fraction of cells could adapt (Figs. S1a, b for 20 mM *3AT*). Nevertheless, the final number of mature adapted colonies, within the range of *3AT* allowing adaptation, was significant and represented a sizable fraction of the population. Adaptation evidently occurred *simultaneously* in many cells in the population and was not due to selection of a special rare subpopulation [24]. Note that higher *3AT* concentrations caused higher variability in colony sizes on plates, indicating higher dispersion in adaptation dynamics and time scales (Fig. S2). As a control, Fig. S3 shows that “wild-type” cells (the same strain with *HIS3* under its native promoter in its native locus), were almost insensitive to the concentration of *3AT* within the range used in the above experiments for the rewired cells, which by contrast showed great sensitivity to the amount of HIS3p’s inhibition. Thus, the response of the cells to *3AT* reflected the challenge due to the cell-cycle’s promoter rewiring. The rewired p*Swi5*-*HIS3* strain proved to be markedly different from the other strains; it could accommodate larger concentrations of *3AT* (20 mM) and remarkably, it eventually grew more colonies on agar plates with 8 mM than on 4 mM of the inhibiting drag, or even more than on a substrate lacking *3AT* (Fig. S1c). This latter point indicates that adaptation to the inhibition of the rewired metabolic protein might work, under certain circumstances, to allow eventually a better tuned set-point of gene regulation in the challenging environment [19].

In principle, the chemostat experiments could not exclude the possibility that the source of the observed variable dynamics between repeated experiments was due to clonal effects, i.e. differences between the specific histories of each population prior to the switch into the *3AT*-containing medium. To test this possibility, we studied the dynamics of “twin” chemostat populations sharing identical histories [30]. Populations with identical histories were created by using two identical chemostat reactors, initiated from a single clone of p*Ndd1-HIS3* cells and coupled via an external pump so that the cells and medium were mixed at a rate much faster than the dilution rate (see *Methods*). A steady state in a *3AT*-free medium was first stabilized for these coupled chemostats, after which the mixing of cells between them was stopped, they were decoupled so that each one contained its own isolated sub-population, and their common feeding medium was supplemented with 4 mM of *3AT*. Thus, after decoupling, the initial single steady-state population was separated into two “twin” populations, allowing the comparison of their separate responses to the environmental pressure. Figure 2b shows that despite originating from a single steady-state mother population, these “twin” populations exhibited different dynamics: one adapted faster than the other by ∼10 generations. Thus, the different growth curves for nominally identical repeated populations mainly reflect intrinsic variability in their adaptation dynamics beyond history-dependent and clonal effects.

Remarkably, the adapted phenotypic state in the *3AT* challenging environment was stably inherited over many generations, as demonstrated by the chemostat dynamics for a p*Swi4*-*HIS3* population in Fig. 2c. In this experiment, removing the *3AT* from the chemostat medium and introducing it back at a later point in time, caused a negligible effect on the population growth dynamics. However, increasing the *3AT* concentration from 4 mM to 8 mM in the same experiment caused a weak declining response in the population density followed by fast re-adaptation. Similar inheritance characteristics were demonstrated for pSwi4-*HIS3* cells adapting on agar plates, as well as for the other two strains, p*Ndd1*-*HIS3* and p*Swi5*-*HIS3* (Figs. S4, S5, and S6) and thus were not a particular feature of the chemostat nor of a specific rewired node of the network. The plate experiments showed that the exposure to a certain amount of *3AT* stabilized a new phenotypic state for the adapted cells, allowing them to accommodate faster and in larger fractions to a growing concentration of *3AT* in the second round of plating (Figs. S4, S5, and S6 b and c). Note again the sensitivity of the inherited response to the concentration of *3AT* in which the original adaptation took place (compare Figs. S4, S5, and S6 b and c). Note that the observed inheritance of the capacity of cells to grow in the challenging 3AT environment proves that this phenomenon represents a genuine adaptation process. It excludes transient intracellular adjustments (e.g., the export of the toxic 3AT out of the cell) or commonly observed stress responses (see the discussion below in connection to the expression response). The inheritance of the adapted state is particularly intriguing since, as was discussed above, the adaptation of numerous individual cells into mature colonies on agar plates testified that adaptation was not due to selection of a rare, advantageous subpopulation [24].

The adaptation dynamics of all chemostat populations were accompanied by clear morphological changes of their cells. Figure 3a shows a sample of typical optical microscopy images of cells harvested from the chemostats of the three strains of Fig. 2a, at different phases of the dynamics: (i) the initial steady state before the addition of *3AT* (upper row), (ii) at the declining phase following the *3AT* supplement (middle row), and (iii) at the final adapted steady state (lower row). Clearly, the cells of all three strains were highly stressed during the declining phase of the chemostat dynamics, as manifested in their change of morphology, proving again that the populations faced a severe challenge due to the inhibition of HIS3p (Fig. 3a middle row). Note the appearance of large vacuoles during the transient phase, a clear indication of stress [31]. Additionally, the pSwi5-HIS3 strain showed cells with shmoo-like structures, reminding a response of yeast to the mating pheromone [32]. Nevertheless, the recovery of the populations after adaptation showed relaxed cells at the steady state phase of the chemostats (Fig. 3a lower row), with similar morphologies to the original steady-state populations (Fig. 3a upper row). Histograms summarizing the cell-size distributions based on analysis of such images are shown in Figs. 3b–d. All strains exhibited a significant shift to a larger size distribution during the transient phase and a shift back to smaller size distributions at the final steady state compared to the ones before the addition of *3AT*. The only exception was the p*Swi*5-*HIS3* strain which exhibited a final size distribution shifted to larger values compared to the initial steady state. But even for this strain, the transient response to *3AT* showed the largest cell sizes, indicating a recovery in cell morphology upon adaptation. Significant modifications in cell size are usually attributed to the functionality of the cell cycle and its proper progression through the various growth phases [32]. Thus, the morphological changes observed in the chemostat experiments suggest that adaptation was also manifested in accommodation of the cell cycle regulation and indicates that pSwi5 is a more plastic node of the network compared with pSwi4 and pNdd1. The greater plasticity of pSwi5 is consistent with its different adaptive capabilities on agar plates discussed above (see Fig. S1).

**Figure 4:**
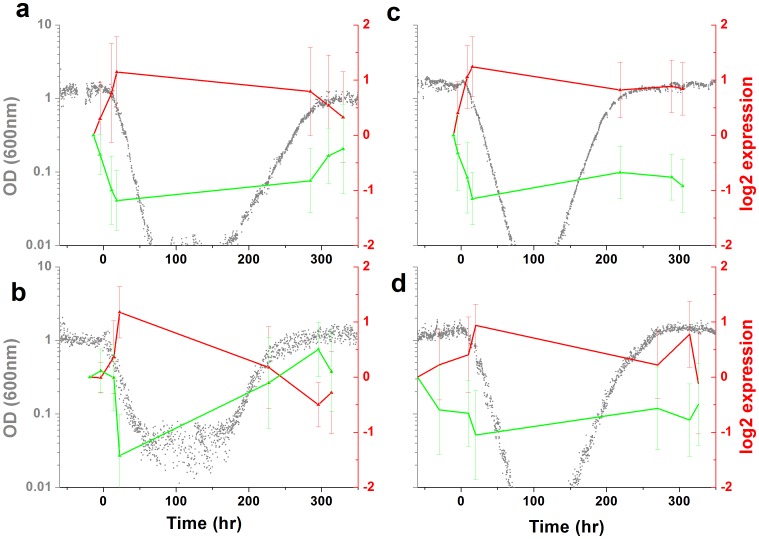
Genome-wide mRNA levels. mRNA was extracted from cell collected from the chemostat experiments at different time points and measured by DNA arrays (see *Methods*). Two major clusters were found using a self-organized maps clustering method, with induced (red) and repressed (green) genes with 2-fold or more compared to the initial steady-state expression levels for: (a) pNdd1-*HIS3* strain with 1051 genes induced and 835 repressed; (b) a “twin” chemostat to that presented in (a) with 526 genes induced and 618 repressed; (c) pSwi4-*HIS3* strain with 616 genes induced and 562 repressed; (d) another chemostat with the pSwi4-*HIS3* strain with 464 genes induced and 476 repressed. The expression levels are presented in log2 values. The error bars represent the standard deviation of expression values among genes belonging to each cluster. The population growth dynamics as measured by the cell density in the chemostats is depicted by the gray curves.

**Figure 5:**
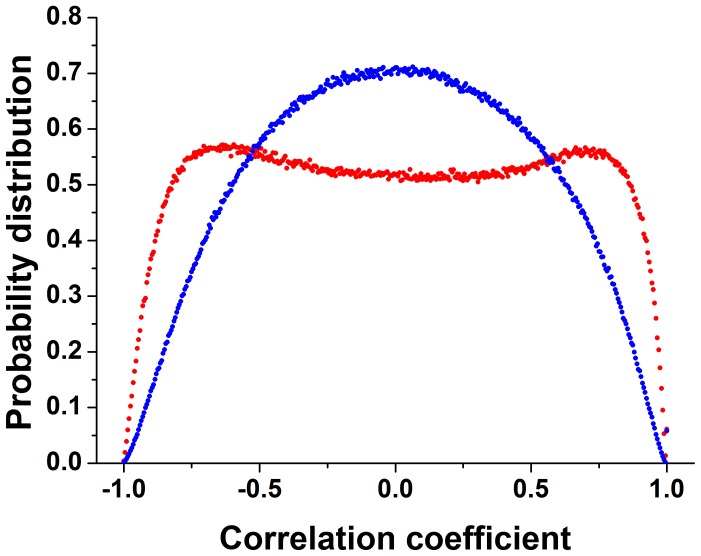
The correlation of transcriptional response. A Pearson correlation coefficient was computed between all pairs of genes based on the array measurements presented in Fig. 4. The figure shows the distributions of correlation coefficient between all possible pairs for the experiment shown in Fig. 4a (pNdd1-*HIS3* strain, blue curve) and Fig. 4c (pSwi4-*HIS3* strain, red curve). The histograms are normalized to unit area and thus represent the probability density.

Next, we investigated the gene expression response underlying the adaptation process. The genome-wide mRNA expression levels of cells harvested from the chemostat at different time points, for two strains with pNdd1-*HIS3* (Figs. 4a,b) and pSwi4-*HIS3* (Figs 4c,d), were measured using DNA arrays (*Methods*). These measurements show that a genome-wide expression response accompanied the adaptation process: a sizable fraction of the genes in the genome changed their expression levels by twofold or more. Cluster analysis revealed two large gene groups responding at the onset of exposure to the *3AT* challenge, one induced and the other, of approximately the same size, repressed (see also Fig. S7). As mentioned above, *3AT* by itself does not cause side effects beyond the specific local inhibition of HIS3p [25], [26], [27] and thus was not the direct cause of these gene expression responses [19], [25]. Comparing the gene expression measurements in repeated experiments showed that the gene content of the responding gene clusters, as well as the number of responding genes, were non-reproducible and varied between nominally identical experiments (Figs. S8–S9). The Pearson correlation coefficient between all the responding genes (>2 fold) in the populations, for two repetitive experiments and even for “twin” populations having identical histories (Figs. 4a,b), showed relatively low values, both during the steady state growth after adaptation (Fig. S8) and during the transient dynamics (Fig. S9). By contrast, each population exhibited significant correlations between its responding genes at two separated time points, both during steady (Fig. S8c) and transient (Figs. S9c,d) growth conditions. The low correlations in transcriptional response between repeated experiments indicate that indeed the patterns of responding genes reflect a non-specific and irreproducible global process [25], [30]. Nevertheless, within a population, the transcriptional pattern was stable and showed high coherency between genes throughout the adaptation dynamics and along steady growth in the adapted state. It indicates the possibility that the underlying response of gene expression reflects collective population dynamics [25], [30]. Obviously, a direct, superficial comparison between the clusters of responding genes for the different strains did not reveal any systematic features that could serve as signatures for an adaptation process that is specific to a particular cell-cycle rewiring event. Tables S1–S2 provide the gene content of the induced and repressed clusters of Fig. 4. This analysis shows that, indeed, the gene content was not reproducible over repeated experiments, except for a small percentage of genes that did not match any particular functional group. The annotation search also revealed that some of the clusters contained significant GO groups, while others did not. The results here are compatible with our previous experiments with HIS3 rewired to the GAL system, that had also demonstrated that the gene expression response does not overlap the common stress responses in yeast (see Table S3 for detailed comparison) [25], [33]. However, as shown in Fig. 5, the rewiring load on the different nodes of the cell cycle network had a clear and distinctive influence on the distribution of the genome-wide pair correlation coefficients, estimated from the array data for the different strains. It revealed that the pSwi4-*HIS3* strain exhibited a unimodal distribution (with mean value at zero; Fig. 5, blue), while the pNdd1-*HIS3* strain showed a bimodal distribution (Fig. 5, red curve. The latter pattern is consistent with stronger correlations between activating and repressing mechanisms compared with the former one. In both cases, however, numerous gene pairs exhibited high correlations, indicating again a global process across functional modules [25].

**Figure 6:**
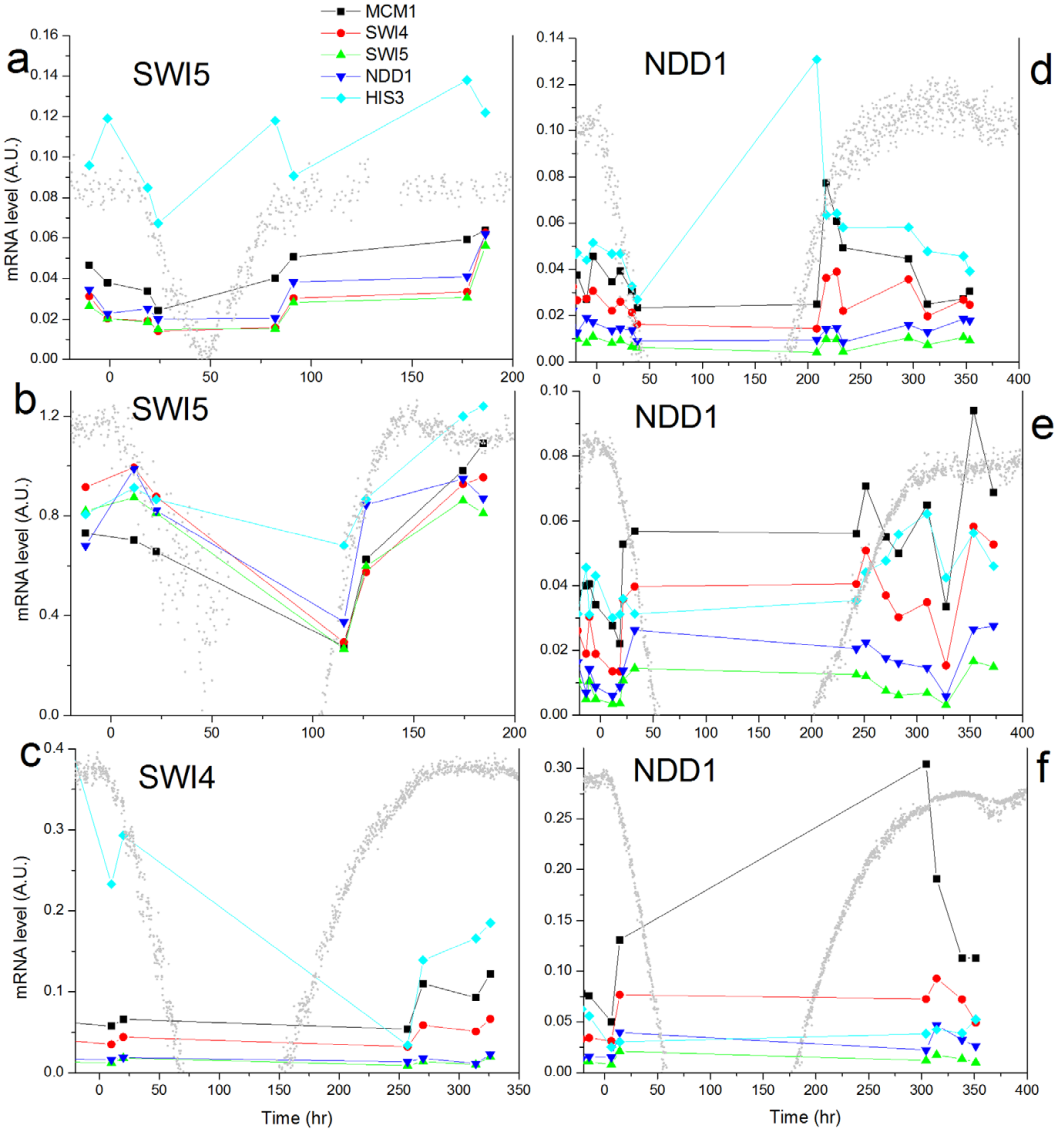
Comparison of mRNA cell-cycle genes expression response. Real-time PCR measurements of the mRNA expression levels for *MCM1* (black), *SWI4* (red), *SWI5* (green), *NDD1* (purple) and *HIS3* (cyan). The measurements were done on cells collected from the same chemostat experiments as in Figure 2, for strains pSwi5-*HIS3* (a,b), pSwi4-*HIS3* (c), and pNdd1-*His3* (d-f). Note that a and b are repeated experiments, d and e are “twin” chemostats decoupled just prior to the switch to 3AT and f is a repeated experiment. The results are average over duplicate measurements (see error-bars in Fig. S10). The mRNA expression levels are normalized by the expression of *ACT1*. The gray curve in each figure is the OD trace of the corresponding population dynamics.

The mRNA expression response of the different strains was studied also at higher precision by real-time PCR. Figure 6 shows the response of some cell cycle genes and *HIS3* for the different strains in six different chemostat experiments. Analysis of additional genes belonging to the cell cycle, histidine and purine pathways is shown in Fig. S10. Results of the real-time PCR on the SWI4, SWI5 and NDD1 rewired strains demonstrated that they did not respond in a significantly different manner. Thus, there was no significant, direct, signature on the transcriptional response of the cell cycle genes. These results are consistent with the observation that adaptation involved a non-specific large-scale transcriptional response. It shows that the robustness of the cell cycle system is manifested through its capability to redistribute the initially local perturbation into a global cellular response. Note that the lack of reproducibility in response between repeated experiments, even between “twin” chemostats (*NDD1*, Fig. 6d,e) is consistent with the results of the array experiments shown in this work, and with our previous work on the GAL system [25], [30].

## Discussion

We have demonstrated that cell populations with rewired genomes, harnessing the cell cycle promoters to regulate an essential metabolic gene, could accommodate a significant inhibition of this protein and rapidly adapted to stabilize a new phenotypic state. Subsequently, this adapted state was stably inherited over generations. A large fraction of the population was shown to be able to adapt on plates and grew into mature colonies, proving that adaptation was not a property of a special rare subpopulation. The adaptation process was accompanied by a non-specific genome-wide transcriptional response exhibiting high correlations between hundreds of genes that reside across the entire metabolic network. The dynamics and list of responding genes were irreproducible over repeated experiments and even between “twin” populations having identical histories. The population adaption capabilities scaled with the *3AT* concentration and different cell cycle promoters exhibited disperse susceptibilities to the challenge, in sharp contrast to the insensitivity of “wild-type" cells. Taken together, we conclude that adaptation involved the emergence of novel regulatory feedbacks between the metabolic system and the cell cycle network. This regulatory plasticity enables the cells to control their growth and reproduction by accommodating to the environmental pressure. It shows that the cell cycle network, despite of being a well conserved and tightly regulated module is an integral part of the cellular plasticity allowing dynamic reorganization of its regulatory modes.

A rapid inherited adaptation of a sizeable fraction of the population accompanied by non-specific and non-reproducible genome-wide expression response also characterized the adaptation of rewired cells with *HIS3* exclusively regulated by the GAL system upon a medium switch from galactose to glucose, from inducing to repressing conditions of the GAL system [19], [24], [25], [30]. This shows that rewiring of an essential metabolic gene to a foreign regulatory system and exposing the cells to an unforeseen challenge stimulates the emergence of a general cellular mechanism allowing a plastic cellular response and rapid adaptation. The case of the cell cycle is different in certain important aspects from that of the GAL system. While the GAL system does not have any particular functional role in glucose and is certainly not essential in this medium [19], the proper functionality of the cell cycle is absolutely essential under all environmental conditions. Thus, while the resources of the GAL system could be fully harnessed to meet the metabolic requirements due to rewiring [19], this is not the case for the cell cycle which must operate in a multi-tasking mode. Moreover, in the case of the cell cycle, each rewired promoter binds a number of transcription factors thus requiring combinatorial re-distribution of resources. By contrast, the GAL system is regulated by a promoter binding a single transcription factor (*GAL4*), the activity of which is modified by interacting with other proteins (e.g., *GAL80*). The cell-cycle rewiring experiments presented here also add a temporal constraint to adaptation that does not exist in the case of the GAL system. Since the transcription factors driving the cell-cycle are active only during specific phases and must be sharply degraded outside of these relatively narrow time windows, their rewiring to control an essential metabolic process add further constraints on their functionality. Furthermore, in native cells these transcription factors are mostly expressed at low levels [18], so that the multitasking challenge is expected to result in a costly re-distribution of relatively limited resources. Thus, the unforeseen rewiring challenge either forces the cell cycle transcription factors to operate outside of their natural context (e.g. outside of their narrow time-window activity), or causes the rewired cells to utilize epigenetic processes to differentiate between the native cell cycle promoter and the one that regulates *HIS3*. In either case, adaptation to genome rewiring in the experiments here proves the highly robust dynamics and flexible interface of the cell cycle network with the cell metabolic system. We observed a significant difference in response between strains with different rewired promoters. In general, pSwi5 exhibits the higher plasticity compared to pNdd1 and pSwi4, as demonstrated by the cell morphology during adaptation (Fig. 3) and its ability to adapt with higher amounts of HIS3p inhibitor on plates (Fig. S1 and Fig. S6). Thus, the genome-rewiring methodology enables a comparative study of the susceptibility and plasticity of different regulatory modules.

*HIS3* does not have a redundant pathway in the budding yeast. Consequently, adaptation to accommodate high levels of its inhibition under the regulation of a cell-cycle promoter required reorganization of the regulatory system enabling its expression at proper levels. This reorganization is manifested in the genome-wide transcriptional response observed in the present experiments. This large-scale transcriptional response necessarily results in metabolic flux re-distributions. The similarities in the adaptation response of cells with *HIS3* rewired to the GAL system [19], [24], [25] and the cell-cycle promoters discussed here reveal that cells can operate a very general and non-specific “search” mechanism. This allows dynamic modifications of their regulatory interactions and supports cellular plasticity and evolvability [19], [34], [35]. The mechanism underlying the inherited adaptation, observed in our experiments is not currently understood and at this stage we cannot identify or exclude specific genetic and epigenetic mechanisms. As in the case of rewiring the GAL system, we cannot exclude the possibility that mutations play a role in the process (see the discussion of this point in [24]). It also remains to be seen how general this phenomenon is in other cell types and in particular in multicellular organisms [36]. It would also be of much interest to utilize genome rewiring as a more general methodology to probe the intrinsic dynamics and plasticity of cell populations. This may be done by linking a wider spectrum of metabolic and other functional genes to foreign promoters and creating conditions under which the modified cells encounter severe unforeseen challenges. Genome rewiring thus manifests itself as a significant class of genomic perturbations, complementary to the more common perturbations in the form of gene deletion or mutations in coding regions. Genome rewiring, however, is of a different nature compared with these latter perturbations; it modifies the regulatory constrains of the genome, and requires the reorganization of regulatory modes to accommodate the challenge rather than production of new functional proteins. Thus, it might serve as a probe of epigenetic processes and dynamics.

In summary, cell adaptation to unforeseen challenges due to genome rewiring turns out to be a general cell property supporting the view that plasticity of the regulatory system plays a significant role in evolution. The cell cycle system proved to be a natural integral part of the adaptable cellular web of gene regulation.

## Materials and Methods

### Strain Construction

Experiments were carried out with the haploid yeast strain YPH499 [Mat *a*, ura3-52, lys2-801, ade2-101, trp1-Δ63, his3Δ200, leu2Δ1]. his3**Δ**200 is a deletion that removed the entire HIS3-coding region as well as the upstream promoter region. Three strains were constructed by the homologues recombination method [37]. The *HIS3* gene was integrated at the *HO* locus under the regulation of pSwi4 (−675 bp to −1 bp of *SWI4* ORF), pNdd1 (−309 bp to −1 bp of *NDD1* ORF) and pSwi5 (−465 bp to −1 bp of *SWI5* ORF) promoters. Cloning was done by standard methods and confirmed by fragments analysis and by direct sequencing. The cloning was based on a commonly used genetic tool in S. *cerevisiae*, using drug resistance markers for G418 and hygromycin B [37]. First, the selected promoters and the *HIS3* ORF sequences were amplified by PCR. The drug resistance cassettes were amplified from pAG32 and pUG6 plasmids (Euroscarf, Germany). Second, the PCR products were fused, using a PCR fusion reaction to have *HIS3* ORF fused with the G418 resistance gene and the cell cycle promoter with the hygromycin one. Finally the yeast cells were transformed with the fused cassettes using lithium acetate method allowing homologous integration into the *HO* locus.

### Chemostat Growth Conditions

Cells were grown in a homemade chemostat [19] in synthetic dropout medium lacking histidine, with the appropriate amino-acid supplement and 2% of pure glucose as the sole carbon source. Medium composition was: 1.7 g/liter yeast nitrogen base without amino acids and ammonium sulfate, 5 g/liter ammonium sulfate, 1.4 g/liter amino acids dropout powder (without tryptophan, histidine, leucine, and uracil; Sigma), 0.01 g/liter l-tryptophan, and 0.005 g/liter uracil and 0.015 g/liter leucine. Growth in the chemostat was limited by the concentration of the amino-acid supplement. To increase the load on HIS3p, the competitive inhibitor *3-amino-1,2,4-triazole* (*3AT*, Sigma), sterilized by filtration, was introduced into the feeding medium. The 4 mM concentration of *3AT* for the chemostat experiments were chosen by first analyzing the growth of the three strains in batch experiments with different concentrations of *3AT*. An online measurement system was used to measure the OD of cells in the chemostat [19]. A cell collector was used to automatically collect samples of cells from the chemostat at precise time points along the experiment and instantaneously freeze them [19]. These samples were used for the microarray experiments. Typical populations in the chemostat contained 10^9^–10^10^ cells. The chemostat dilution time was ∼7 hrs which translates into 7×ln2∼5 hrs generation time.

### “Twin” Populations

Two identical chemostats were started from a single clone of the pNdd1 strain and operated in parallel. The two chemostats had a closed-loop line between them, allowing fast mixing of the cells via a separate pump [30]. Steady state in glucose medium lacking histidie was established while mixing was done at a fast rate. Since the mixing of cells between the coupled chemostats at this stage was much faster than their dilution rates, as long as they were coupled they effectively contained a single population: the fast mixing caused the same cells to pass several times back and forth between the reactors before being diluted out. The mixing line was decoupled prior to the addition of *3AT* to the medium. The feeding source was common to the two chemostats throughout the experiment. Each chemostat had its own OD online measurement system and homemade cell collector.

### Plates Growth Conditions

Agar plates were made with 0.04 g/liter l-tryptophan, 0.02 g/liter uracil, 0.06 g/liter leucine, 2% glucose, and 2% agar. The plates’ media were supplemented with different concentrations of *3AT*: 0,4,8, 12 and 20 mM. In adaptation experiments, a starter of cells from each strain was propagated in a batch for 48 h in 30°c in the same medium as used for the plates without *3AT*. Cells from the batch culture with OD (600 nm)∼1 were distributed on plates with the aid of pretreated sterile glass beads and incubated at 30°c. Colonies were counted manually and images of the plates were taken. In the inheritance experiments of Figs. S3, S4, and S5, one adapted colony from each clone from the plates with 4 or 8 mM *3AT* was suspended, diluted and then re-distributed on plates with concentrations of 0,4,8 and 12 mM *3AT*.

### Microscopy Analysis

Imaging was done with an inverted microscope (Zeiss Axiovert 135) with 100× oil-immersed objective. The bright field images were used to identify cell boundaries using ImagePro software (Media-Cyberentics). Further analysis to measure the areas of Cells was performed by homemade software written in Matlab. Histograms were constructed from several hundred cells each, and normalized for unit area.

### Expression Arrays

mRNA expression measurements followed the same procedures as in ref [25]. They were done for two independent chemostat experiments with the pSwi4-*HIS3* strain, and for a pair of “twin” chemostats (see above) with the pNdd1-*HIS3* strain. Samples of cells were collected from the chemostats along each experiment at 7 different time points. From each sample, 15 µg of total RNA was isolated using hot phenol extraction [25]. mRNA was reverse transcribed (superscript II, Invitrogen) and labeled indirectly with cy5/3 dyes (Amersham) using amino-allyl dUTP (Ambion). For each time point, two cDNA microarrays (yeast 6.4 k, UHN microarray center, www.microarrays.ca) containing all ∼6400 yeast ORFs in duplicate (a total of four spots for each ORF) were hybridized overnight (42°C) with the sample labeled with cy5 and a reference sample labeled with cy3. Arrays were scanned using a commercial scanner and software (GenePix 4000B, Axon instruments). For each microarray, cy5/cy3 intensity ratios were normalized using the Acuty software (Axon instruments), so the ratio of medians was 1. log2(cy5/cy3) values of all spots for each gene were averaged for each time point and only genes with at least two high-quality spots in each time point and full dynamic path along the experiments were subject for further analysis. Duplicate arrays were checked to yield high correlated signal for each gene (Fig. S8d) and the data from each array were compared to real-time PCR measurements for various genes from each sample. Between 3200 and 4200 genes passed all filters in the experiments (two Swi4p and two Ndd1p chemostat experiments). For all analyses the log2(cy5/cy3) values in each time point were normalized to the first steady-state time point.

### Clustering Analysis

Clustering analysis followed the same procedures as in ref [25]. All genes with a two-fold change in at least one time point were clustered using the EXPANDER software [38]. The self-organized maps clustering method [38], [39] was applied to the gene profiles, with 16 clusters as a pre-defined parameter. Clusters that show the same fundamental mean expression profile (Fig. S7) were joined into two large clusters presented in Fig. 4. These results were not sensitive to the self-organized maps clustering method and similar results have been obtained with different methods (data not shown). Enrichment of biological process was computed for clusters of genes using the GO TermFinder (SGD, Tables S1-S2). All P-values were computed using the hypergeometric distribution.

### mRNA Measurements Using Real-time PCR

Total RNA was prepared from cells collected from the chemostats at precise time points, by phenol extraction followed by cDNA preparation (oligo-d(T)16; TAQMAN-Reverse Transcription Kit, Applied Biosystems). Real-time PCR measurements were performed with AB 7700 (SYBR master mix, AB). Measured amounts of ACT1 prepared by PCR served as a ruler. All measurements were normalized by the ACT1 transcription level measured in each sample as the other genes. In all measurements a non-template control for each of the primer pairs resulted in at least two orders of magnitude lower signal. Some of the measurements were performed in duplicates in two separate PCR measurements. Typical measurement errors are shown in Fig. S10. mRNA levels from 18 genes belonging to four different functional groups were measured: cell-cycle: *mcm1, swi4, swi5, clb2, cln2, cln3, whi5, ndd1, sic1*; Histidine pathway: *His1, His3,His4, His6,His7, His5*; Purine pathway: *ynd1*, *pnp1*, *imd4*,.

## Supporting Information

Figure S1 **Adaptation on plates.** Rewired cells were grown in batch cultures in media lacking histidine and *3AT* and dispersed on plates (duplicates) with the same media and different *3AT* concentrations (see *Methods*). The number of mature visible colonies were counted as a function of time (average over duplicates) for 4 mM *3AT* (black curves), 8 mM *3AT* (blue curves) and 20 mM *3AT* (red curves). (a) pSwi4-HIS3 (maximal variability between duplicates ∼16%), (b) pNdd1-*HIS3* (maximal variability between duplicates ∼9%), and (C) pSswi5-*HIS3* (maximal variability between duplicates ∼30%). The lines are spline extrapolations to guide the eye. The number of observed colonies was normalized to the number observed on plates with 0 mM *3AT.* Note that first colonies appeared after a period longer than ∼4 days (for comparison, cells in a medium lacking histidine grow into mature colonies after ∼2 days).
(PDF)

Figure S2 **Images of colonies on plates.** Images of plates after adaptation for: rich media (first column), 4 mM *3AT* (second column), 8 mM *3AT* (third column), and 20 mM *3AT* (fourth column) for the different rewired strains as indicated. Samples from batch cultures were plated as in Fig. S1. For each strain the number of plated cells on all plates was identical.
(PDF)

Figure S3 **The growth of “wild-type” cells under 3AT.** (a) “Wild-type” cells with *HIS3* under its native promoter. Left: no *3AT*; Right: 4 mM *3AT*. The images were captured after 72 hrs. While there is a slight delay in the appearance of visible colonies on *3AT* plates, the overall growth was more or less similar to that with no *3AT*. The medium is similar to the one used in the paper. (b) “wild-type” cells grown with different concentrations of *3AT* (as marked) imaged after 4 days on plates. Note the lack of sensitivity to the 3AT concentration. The table on the right quantifies the number of visible colonies in duplicate plates after 4 days. While there are variations, within the experimental errors there is clearly no sensitivity in the ability of cells to grow to mature colonies to the *3AT* concentration.
(PDF)

Figure S4 **Inheritance of adapted phenotypes.** Cells of the strain pSwi4-*HIS3* were grown in a batch culture with no *3AT* and then dispersed on plates with different concentrations of *3AT* as in Fig. S1 (results repeated in (a)). After adaptation, mature colonies from plates with (b) 4 and (c) 8 mM *3AT* were re-plated for a second phase of growth on plates with 4 mM *3AT* (black curve), 8 mM *3AT* (blue curve) and 12 mM *3AT* (red curve) and the number of mature visible colonies were counted as a function of time (average over duplicates).
(PDF)

Figure S5 **Inheritance of adapted phenotypes.** The same as Fig. S4 for cells of the strain pNDD1-*HIS3*.
(PDF)

Figure S6 **Inheritance of adapted phenotypes.** The same as Fig. S4 for cells of the strain pSwi5-*HIS3*.
(PDF)

Figure S7 **Clustering analysis for DNA arrays.** Only active genes which exhibited at least a twofold change in at least one time point along each of the experiments were subjected to clustering (1645 and 1519 genes out of 3281 and 3446, respectively, for the pSwi4-*HIS3* strain, 1656 and 2433 out of 3266 and 4252, respectively, for the pNdd1-*HIS3* strain). The Self Organizing Maps (SOM) clustering method [39] which is implemented in the EXPANDER microarray analysis package [38] was applied to these gene profiles, with 16 clusters as a pre-defined parameter (the results are not sensitive to the predefined number of clusters parameter implemented in the SOM algorithm). The 16 clusters (overall average homogeneity: 0.72 and 0.76 for the pSwi4-HIS3 strain and 0.86 and 0.76 for the pNdd1-HIS3 strain) show that 57–78% of the active genes in all the experiments exhibited a mean expression pattern of significant induction/repression after the addition of *3AT* to the medium, and then a relaxation on the time scale of cells adaptation. (a) pNdd1-*HIS3* strain with 1051 genes induced (assigned to 7 clusters) and 835 repressed (assigned to 5 clusters); (b) a “twin” chemostat to that presented in (a) with 526 genes induced (assign to 4 clusters) and 618 repressed (assigned to 6 clusters); (c) pSwi4-*HIS3* strain with 616 genes induced (assign to 5 clusters) and 562 repressed (assigned to 5 clusters); (d) another chemostat with the pSwi4-HIS3 strain with 464 genes induced (assigned to 4 clusters) and 476 repressed (assigned to 5 clusters). The expression levels are presented in log2 values. The error bars represent the standard deviation of expression values among genes belonging to each cluster. The population growth dynamics as measured by the cell density in the chemostats is depicted by the gray curves.
(PDF)

Figure S8 **Comparison between the expression levels at steady states of two repeated experiments.** The mRNA levels as measured by the DNA arrays (see *Methods*) comparing the adapted steady state for different chemostat populations for: (a) pSwi4-*HIS3* versus pNdd1-*HIS3* strains; (b) two populations of the pNdd1-*HIS3* strain in “twin” chemostats; (c) two points in time separated by 20 hrs within the steady state after adaptation for the same pNdd1-*HIS3* population; and (d) for comparison, typical array duplicates at the same point reflecting the error in the array experiments. The lines are linear fits to the data and the R values are the Pearson correlation coefficients (linear regression) for each plot. Note the significantly lower correlation for separate experiments compared to time points within the same experiment.
(PDF)

Figure S9 **Comparison between the expression levels in the transient phase for two repeated experiments.** The mRNA levels as measured by the DNA arrays (see *Methods*) at ∼16 hrs after the addition of *3AT* to the chemostat medium comparing two populations. (a) Comparison between two transient points of pSwi4-*HIS3* and pNdd1-*HIS3* strains. (b) Comparison between two transient points of a pair of “twin” pNdd1-*HIS3* populations. (c) Comparison between two transient points separated by 7 hrs of the same pNdd1-*HIS3* strain population. (d) Comparison between transient and steady state points within the same pNdd1-*HIS3* experiment, separated by 390 hrs. The lines are linear fits to the data and the R values are the Pearson correlation coefficients between the experiments on the two axes. Note the negative correlations between two repeated experiments compared to the relatively high positive correlations between two time points within the same experiment.
(PDF)

Figure S10 **mRNA expression response.** Real-time PCR measurements of the mRNA expression levels for genes belonging to: the cell-cycle system (left), histidine pathway (middle), and the purine pathway (right) for cells collected from the same chemostat experiments as in Figure 2 main text. The results are for the following strains: pSwi5-*HIS3* (upper row), pSwi4-*HIS3* (second row) and pNdd1-*His3* for two twin chemostats decoupled just prior to the switch to 3AT (last two rows). The mRNA expression levels are normalized by the expression of *ACT1*. The results shown are the average over duplicate measurements and the error-bars are the standard deviations. The gray curve in each figure is the OD trace of the corresponding population dynamics.
(PDF)

Table S1 **GO term groups for a pSwi4-** ***HIS3*** **population.** Gene content of the expression clusters, as well as the number of responding genes, were non-reproducible and varied between nominally identical experiments. There was 8% matching between the genes in the repressed clusters of the pNdd1-*HIS3* experiments (the “twin chemostats”), and 9% between the induced clusters. In the pSwi4-*HIS3* experiments there were 15% and 10% matching between the genes in the repressed and induced clusters, respectively. Enrichment of biological process was computed for clusters of genes using the GO TermFinder [40], [41]. The annotation search revealed that some of the clusters contained significant GO groups, while other did not. Table S1 depicts the Go term groups in one pSwi4-*HIS3* experiment (in the other pSwi4-*HIS3* experiment no GO groups were found).
(PDF)

Table S2 **GO term groups, pNdd2 experiment.** The same as Table S1 for a pNDD1-*HIS3* experiment (in the other pNDD1-*HIS3* experiment no GO groups were found).
(PDF)

Table S3 **Comparison of the genome-wide mRNA expression in our experimentsto genome-wide expressions in stress response and amino acid starvation.** (a) The fraction of genes in our experiments that showed at least 2 fold change (induced/repressed) of the expression after the addition of 3AT (4th time point) relative to the initial steady-state and were also induced or repressed in the environmental stress response (ESR, Fig. 3 in [33]). It is clear that the overlap between the expression patterns observed in our experiments and that of the known stress response is insignificant. (b), (c) Amino acid starvation expression data was derived from [42]. The gene expression values that were induced/repressed significantly (at least 2 fold change, p<0.05) in their dataset C (+/−100 mM 3AT) have been compared to our expression data (same as in (a)). The table presents the correlation coefficients between the two experiments. It is clear that there is no correlation between the pattern of expression observed in our experiments and the response to amino acid starvation.
(PDF)
