# Supplementary figures and images for: Cellular Plasticity Enables Adaptation to Unforeseen Cell-Cycle Rewiring Challenges
Source: PLoS One. 2012 Sep 18;7(9):e45184. doi: 10.1371/journal.pone.0045184 (PMC3445480; doi:10.1371/journal.pone.0045184)

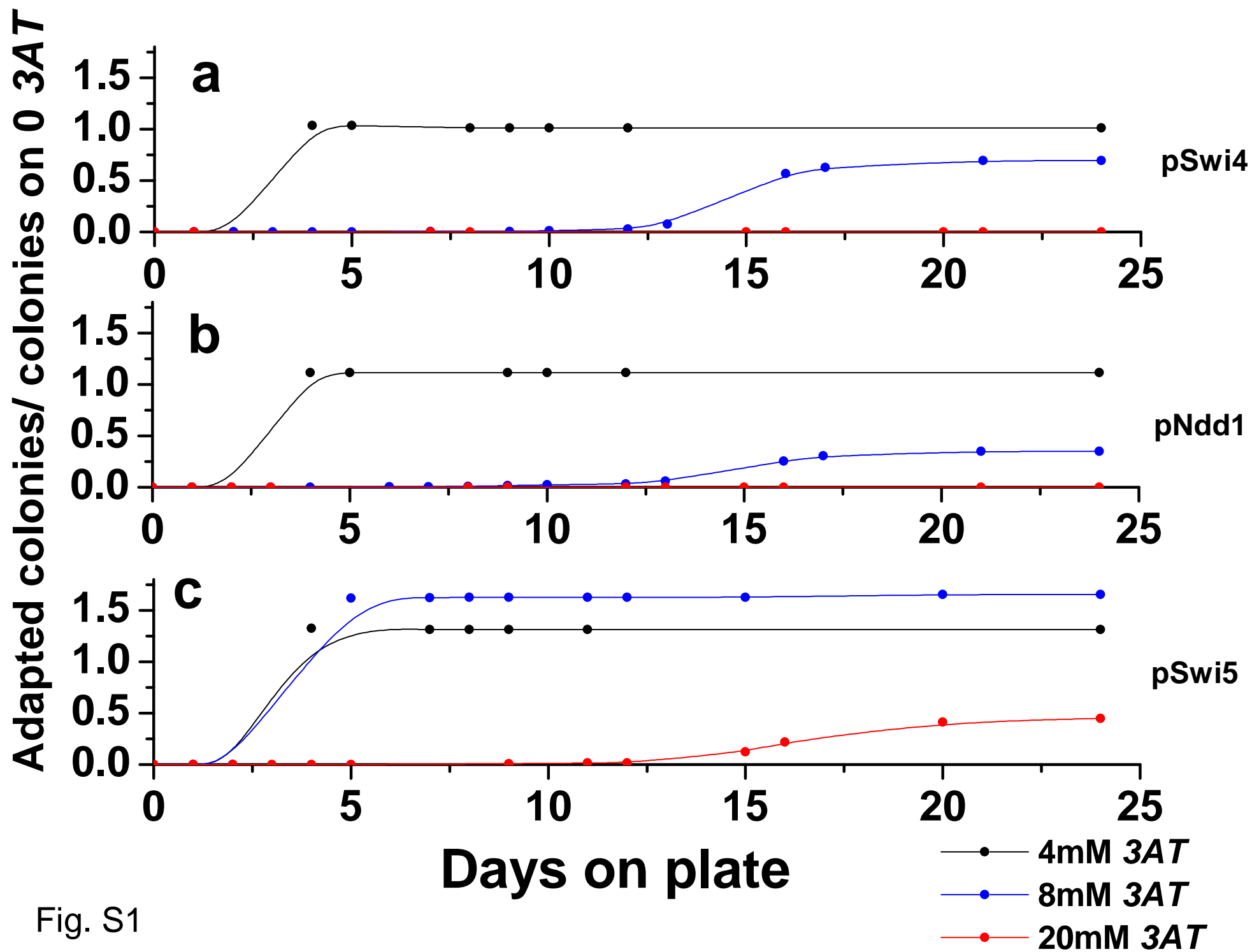

Fig. S1

Supplement: Figure S1 — Adaptation on plates. Rewired cells were grown in batch cultures in media lacking histidine and 3AT and dispersed on plates (duplicates) with the same media and different 3AT concentrations (see Methods). The number of mature visible colonies were counted as a function of time (average over duplicates) for 4 mM 3AT (black curves), 8 mM 3AT (blue curves) and 20 mM 3AT (red curves). (a) pSwi4-HIS3 (maximal variability between duplicates ∼16%), (b) pNdd1-HIS3 (maximal variability between duplicates ∼9%), and (C) pSswi5-HIS3 (maximal variability between duplicates ∼30%). The lines are spline extrapolations to guide the eye. The number of observed colonies was normalized to the number observed on plates with 0 mM 3AT. Note that first colonies appeared after a period longer than ∼4 days (for comparison, cells in a medium lacking histidine grow into mature colonies after ∼2 days). (PDF) [file pone.0045184.s001.pdf]

**Rich medium**

**4 mM 3AT**

**8 mM 3AT**

***pSwi4***

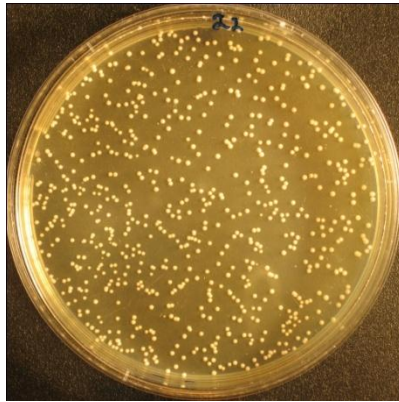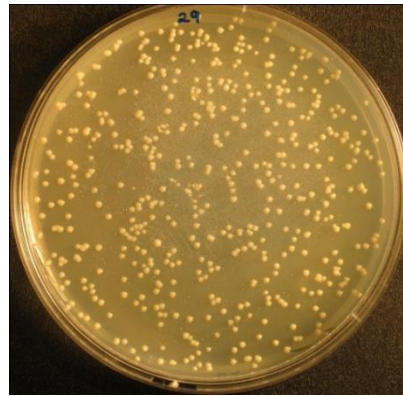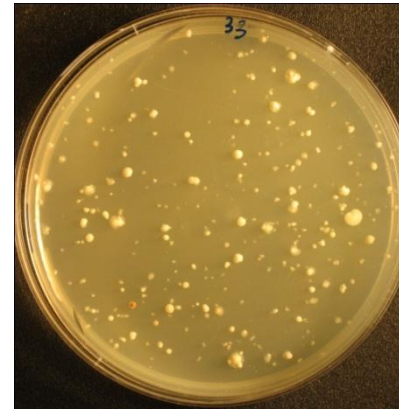

***pNdd1***

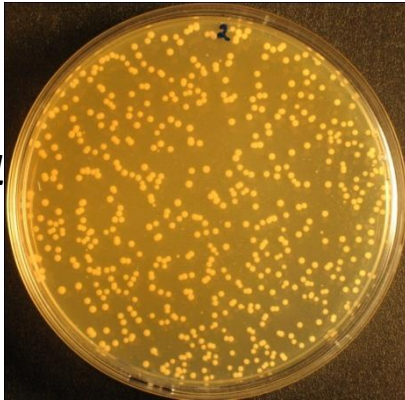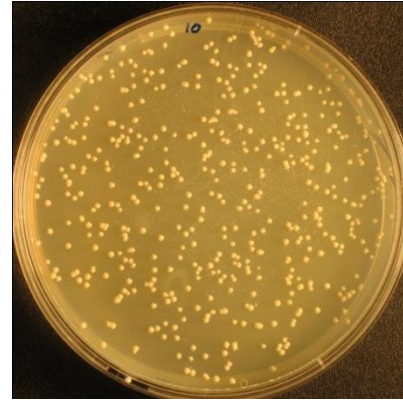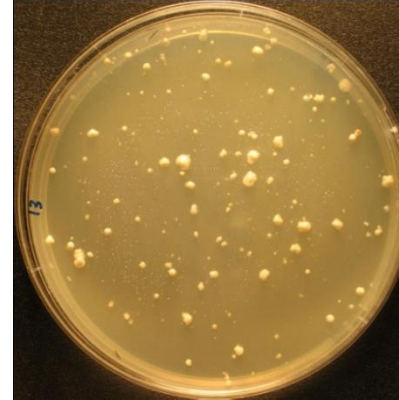

**20 mM 3AT**

***pSwi5***

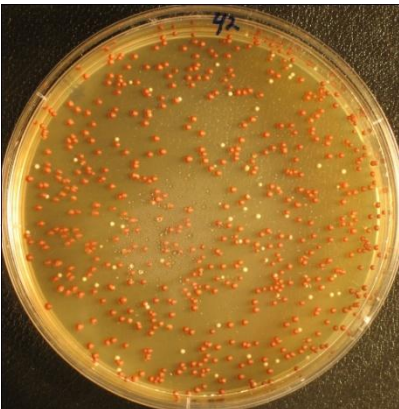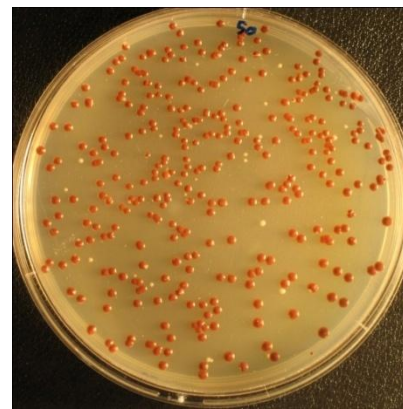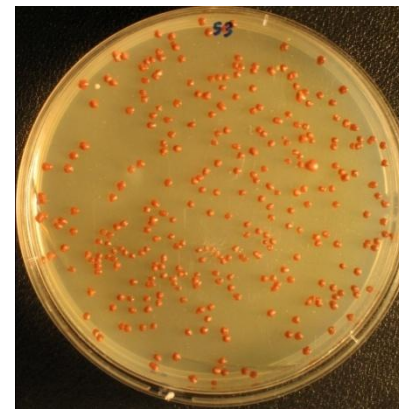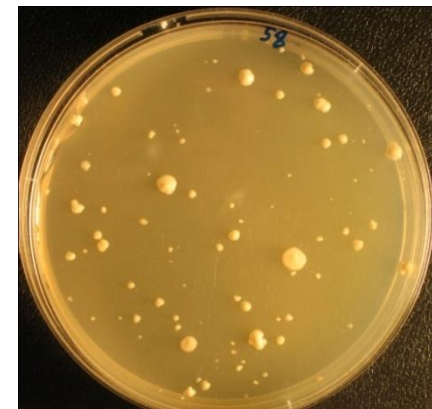

**Fig. S2**

Supplement: Figure S2 — Images of colonies on plates. Images of plates after adaptation for: rich media (first column), 4 mM 3AT (second column), 8 mM 3AT (third column), and 20 mM 3AT (fourth column) for the different rewired strains as indicated. Samples from batch cultures were plated as in Fig. S1. For each strain the number of plated cells on all plates was identical. (PDF) [file pone.0045184.s002.pdf]

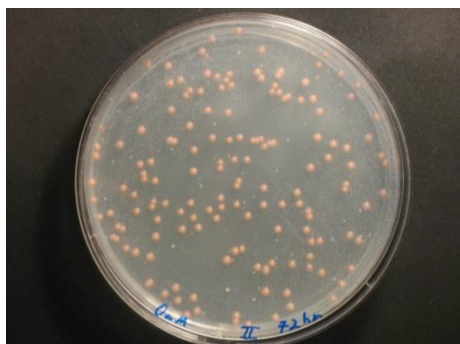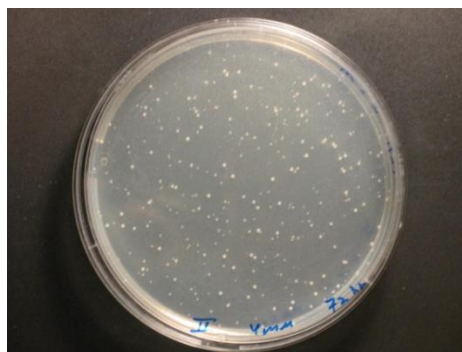

**Fig. S3a**

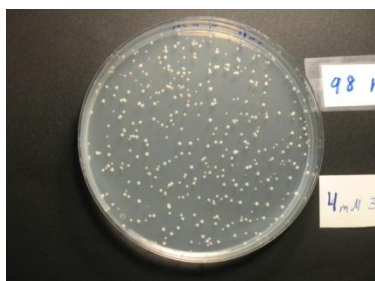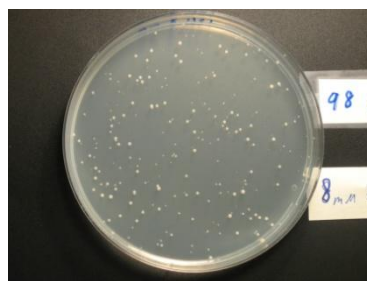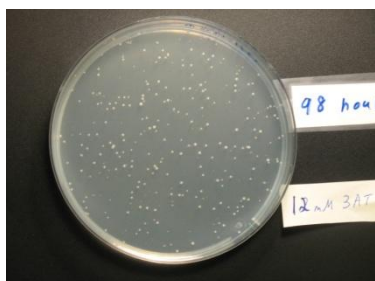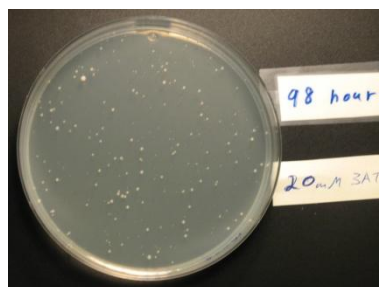

| 3AT<br>(mM) | Pla<br>te I | Pla<br>te II |
|-------------|-------------|--------------|
| 0           | 319         | 258          |
| 4           | 350         | 433          |
| 8           | 323         | 247          |
| 12          | 316         | 238          |
| 20          | 238         | 245          |

**Fig. S3b**

Supplement: Figure S3 — The growth of “wild-type” cells under 3AT. (a) “Wild-type” cells with HIS3 under its native promoter. Left: no 3AT; Right: 4 mM 3AT. The images were captured after 72 hrs. While there is a slight delay in the appearance of visible colonies on 3AT plates, the overall growth was more or less similar to that with no 3AT. The medium is similar to the one used in the paper. (b) “wild-type” cells grown with different concentrations of 3AT (as marked) imaged after 4 days on plates. Note the lack of sensitivity to the 3AT concentration. The table on the right quantifies the number of visible colonies in duplicate plates after 4 days. While there are variations, within the experimental errors there is clearly no sensitivity in the ability of cells to grow to mature colonies to the 3AT concentration. (PDF) [file pone.0045184.s003.pdf]

# pSwi4-*HIS3*

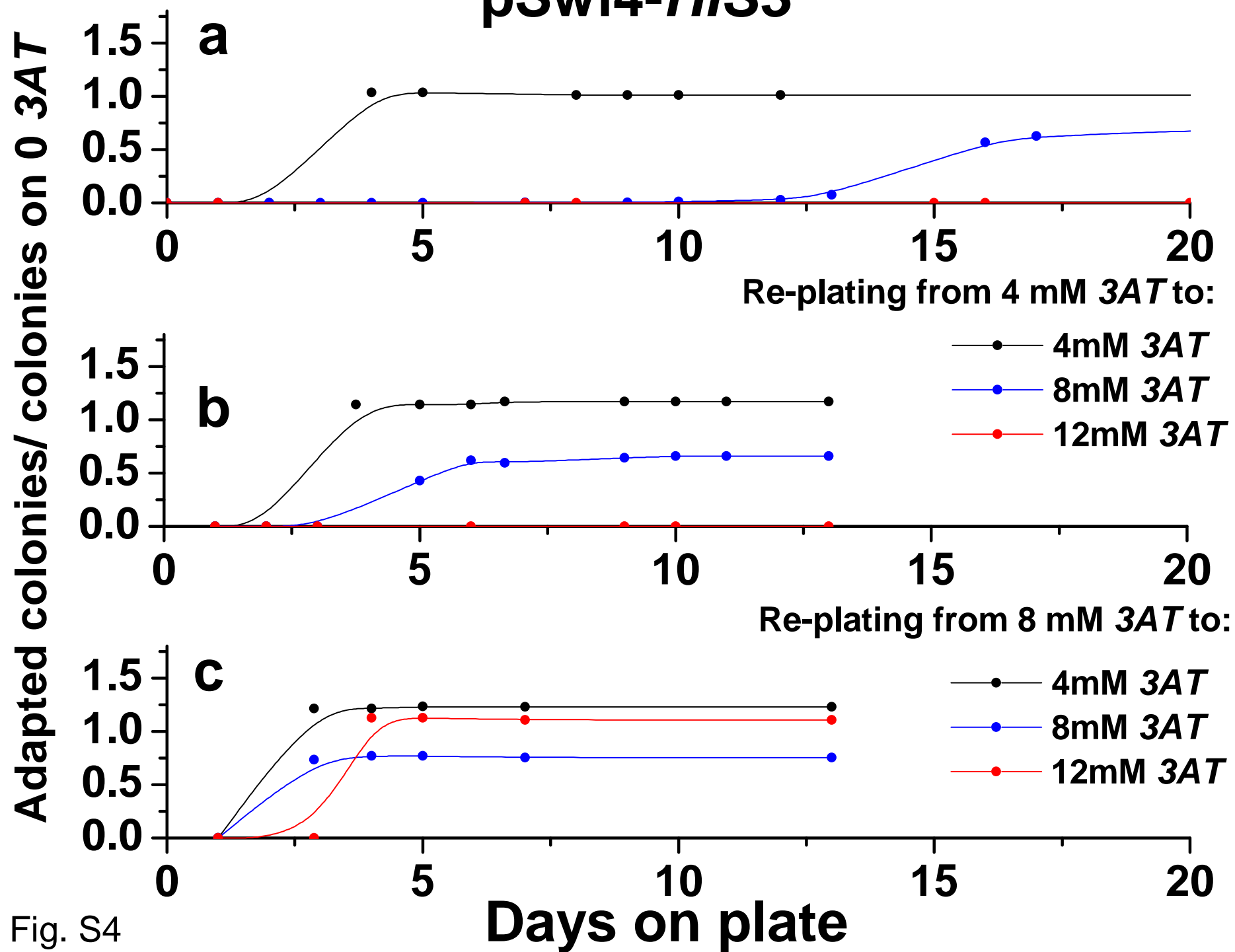

Fig. S4

Supplement: Figure S4 — Inheritance of adapted phenotypes. Cells of the strain pSwi4-HIS3 were grown in a batch culture with no 3AT and then dispersed on plates with different concentrations of 3AT as in Fig. S1 (results repeated in (a)). After adaptation, mature colonies from plates with (b) 4 and (c) 8 mM 3AT were re-plated for a second phase of growth on plates with 4 mM 3AT (black curve), 8 mM 3AT (blue curve) and 12 mM 3AT (red curve) and the number of mature visible colonies were counted as a function of time (average over duplicates). (PDF) [file pone.0045184.s004.pdf]

# pNDD1-*HIS3*

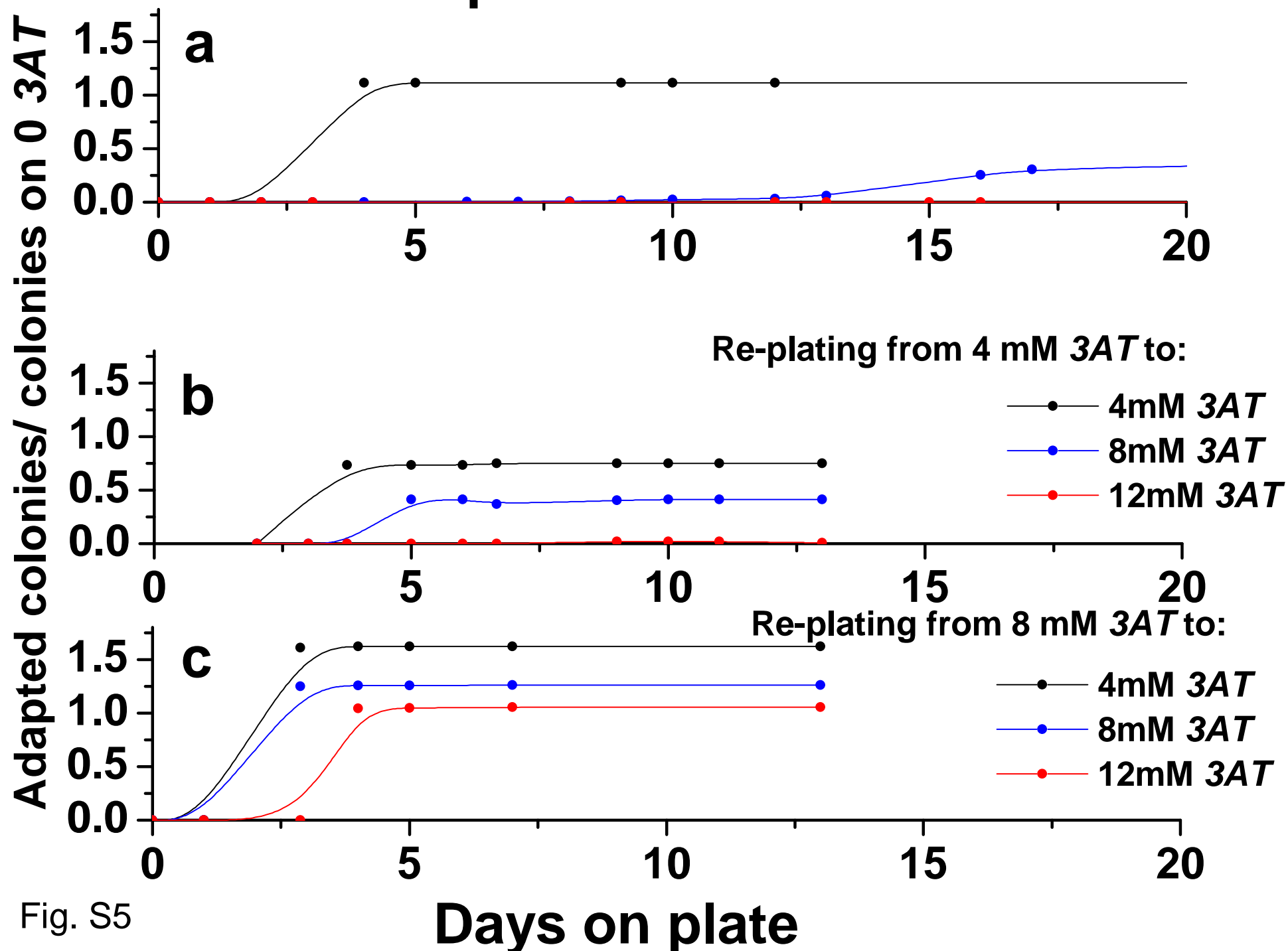

Fig. S5

Supplement: Figure S5 — Inheritance of adapted phenotypes. The same as Fig. S4 for cells of the strain pNDD1-HIS3. (PDF) [file pone.0045184.s005.pdf]

# pSwi5-*HIS3*

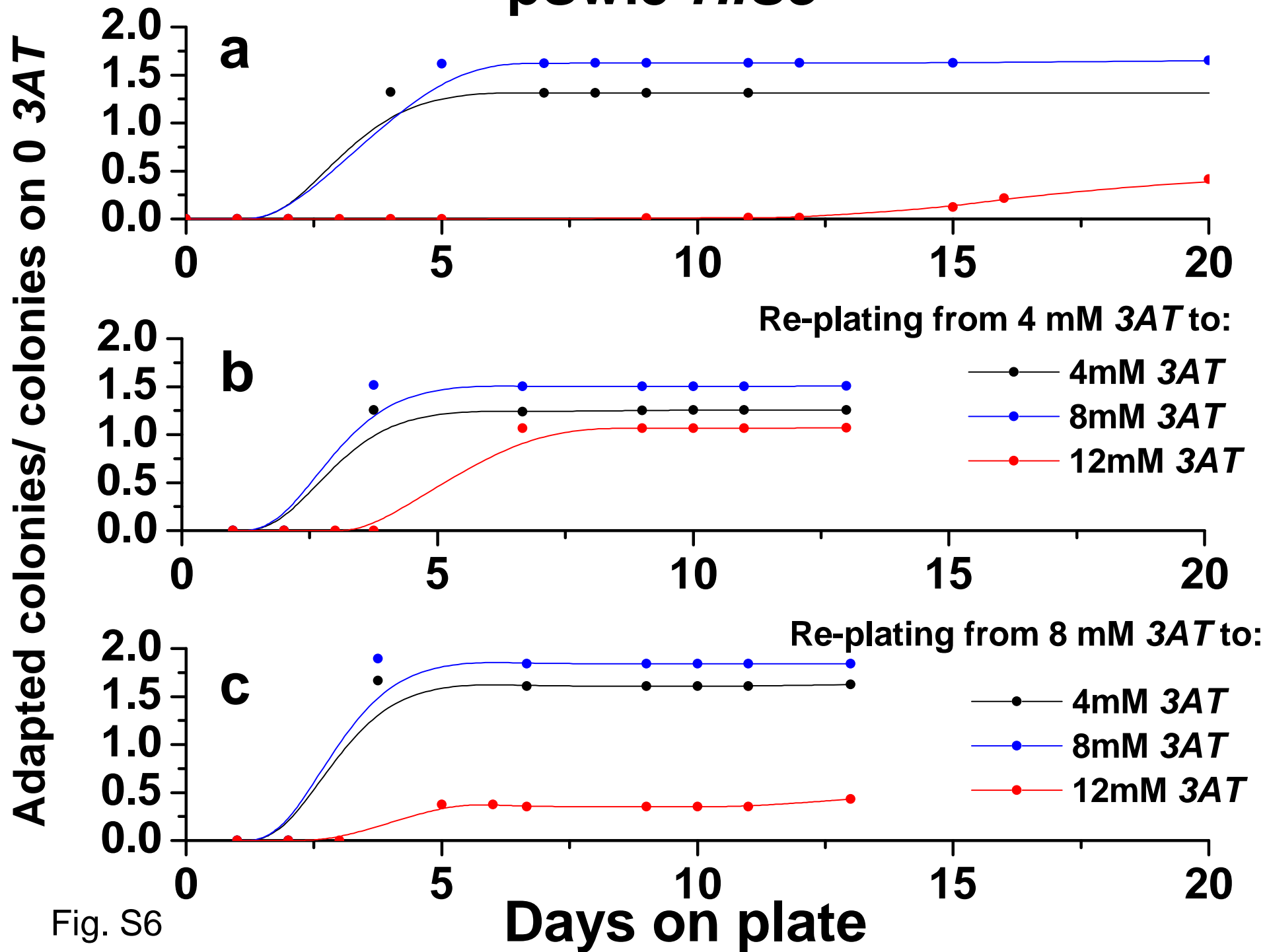

Fig. S6

Supplement: Figure S6 — Inheritance of adapted phenotypes. The same as Fig. S4 for cells of the strain pSwi5-HIS3. (PDF) [file pone.0045184.s006.pdf]

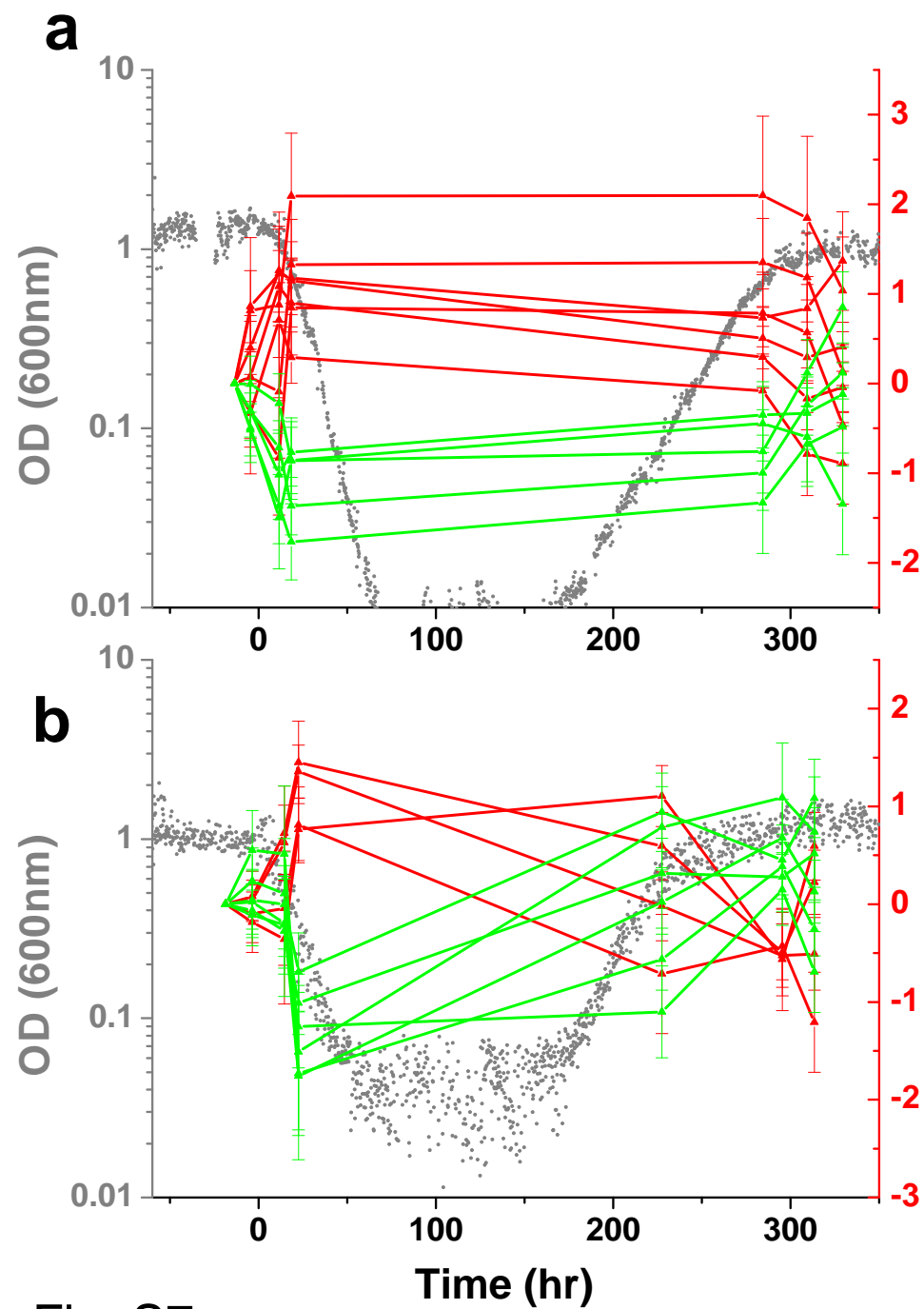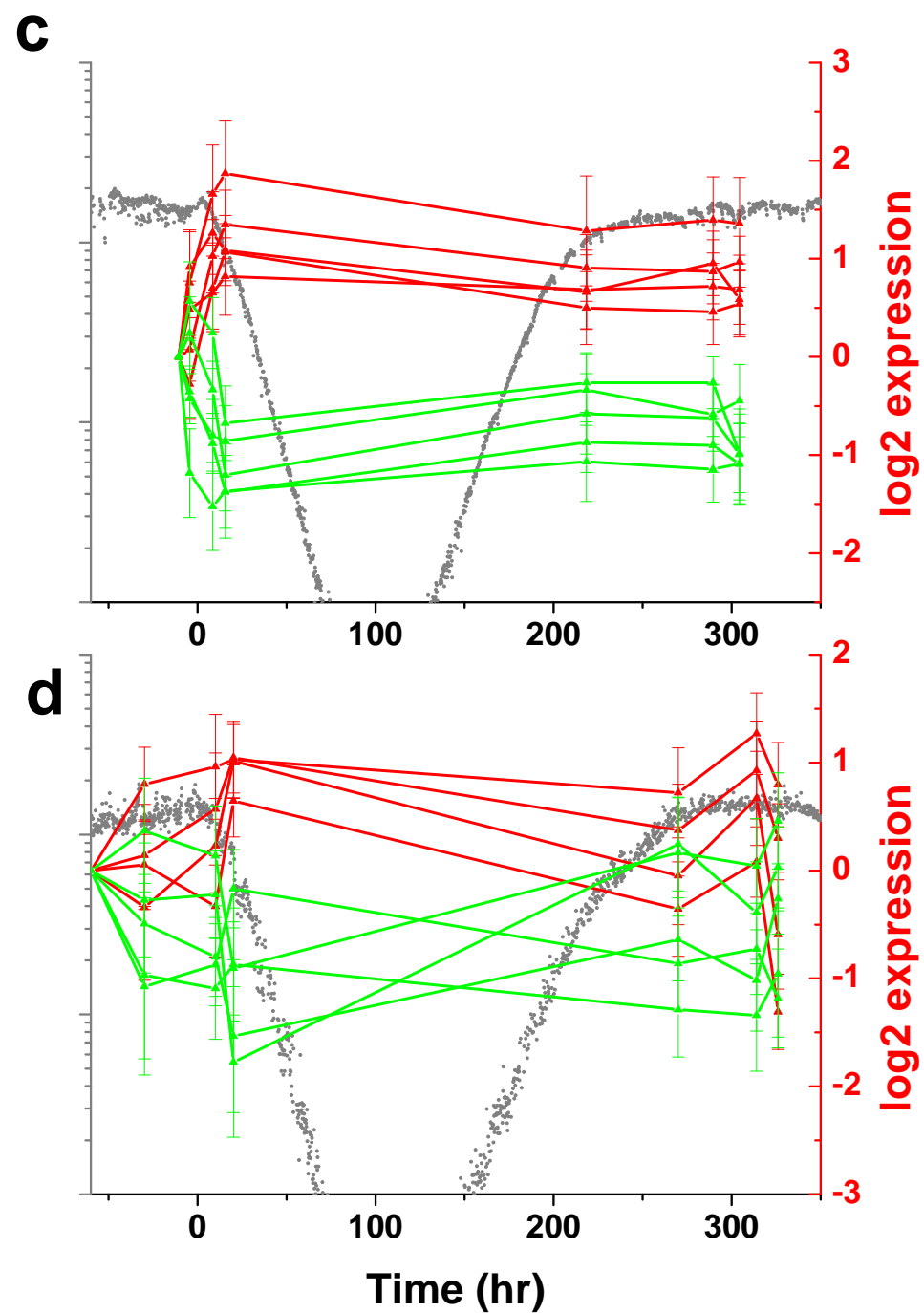

Fig. S7

Supplement: Figure S7 — Clustering analysis for DNA arrays. Only active genes which exhibited at least a twofold change in at least one time point along each of the experiments were subjected to clustering (1645 and 1519 genes out of 3281 and 3446, respectively, for the pSwi4-HIS3 strain, 1656 and 2433 out of 3266 and 4252, respectively, for the pNdd1-HIS3 strain). The Self Organizing Maps (SOM) clustering method [39] which is implemented in the EXPANDER microarray analysis package [38] was applied to these gene profiles, with 16 clusters as a pre-defined parameter (the results are not sensitive to the predefined number of clusters parameter implemented in the SOM algorithm). The 16 clusters (overall average homogeneity: 0.72 and 0.76 for the pSwi4-HIS3 strain and 0.86 and 0.76 for the pNdd1-HIS3 strain) show that 57–78% of the active genes in all the experiments exhibited a mean expression pattern of significant induction/repression after the addition of 3AT to the medium, and then a relaxation on the time scale of cells adaptation. (a) pNdd1-HIS3 strain with 1051 genes induced (assigned to 7 clusters) and 835 repressed (assigned to 5 clusters); (b) a “twin” chemostat to that presented in (a) with 526 genes induced (assign to 4 clusters) and 618 repressed (assigned to 6 clusters); (c) pSwi4-HIS3 strain with 616 genes induced (assign to 5 clusters) and 562 repressed (assigned to 5 clusters); (d) another chemostat with the pSwi4-HIS3 strain with 464 genes induced (assigned to 4 clusters) and 476 repressed (assigned to 5 clusters). The expression levels are presented in log2 values. The error bars represent the standard deviation of expression values among genes belonging to each cluster. The population growth dynamics as measured by the cell density in the chemostats is depicted by the gray curves. (PDF) [file pone.0045184.s007.pdf]

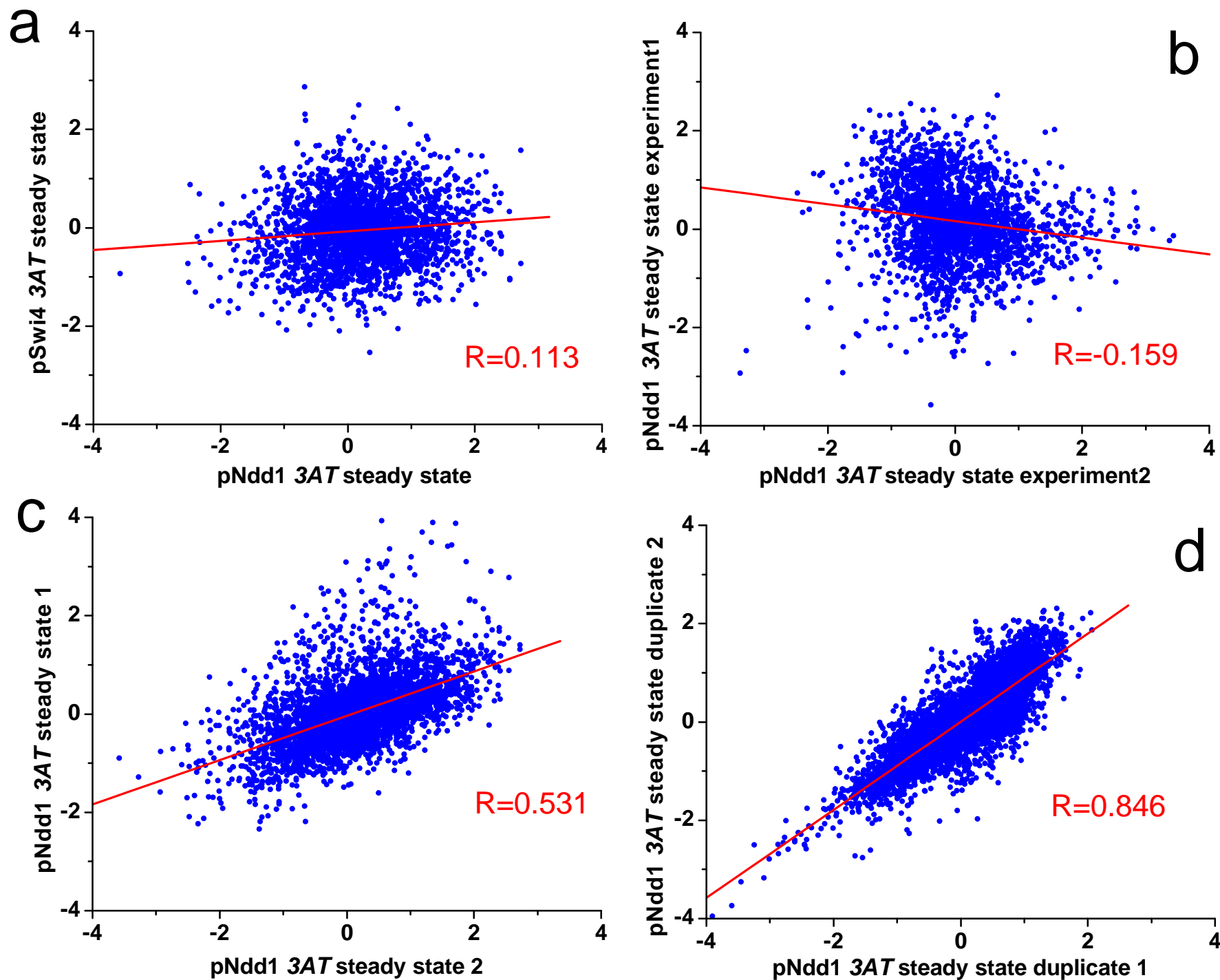

Fig. S8

Supplement: Figure S8 — Comparison between the expression levels at steady states of two repeated experiments. The mRNA levels as measured by the DNA arrays (see Methods) comparing the adapted steady state for different chemostat populations for: (a) pSwi4-HIS3 versus pNdd1-HIS3 strains; (b) two populations of the pNdd1-HIS3 strain in “twin” chemostats; (c) two points in time separated by 20 hrs within the steady state after adaptation for the same pNdd1-HIS3 population; and (d) for comparison, typical array duplicates at the same point reflecting the error in the array experiments. The lines are linear fits to the data and the R values are the Pearson correlation coefficients (linear regression) for each plot. Note the significantly lower correlation for separate experiments compared to time points within the same experiment. (PDF) [file pone.0045184.s008.pdf]

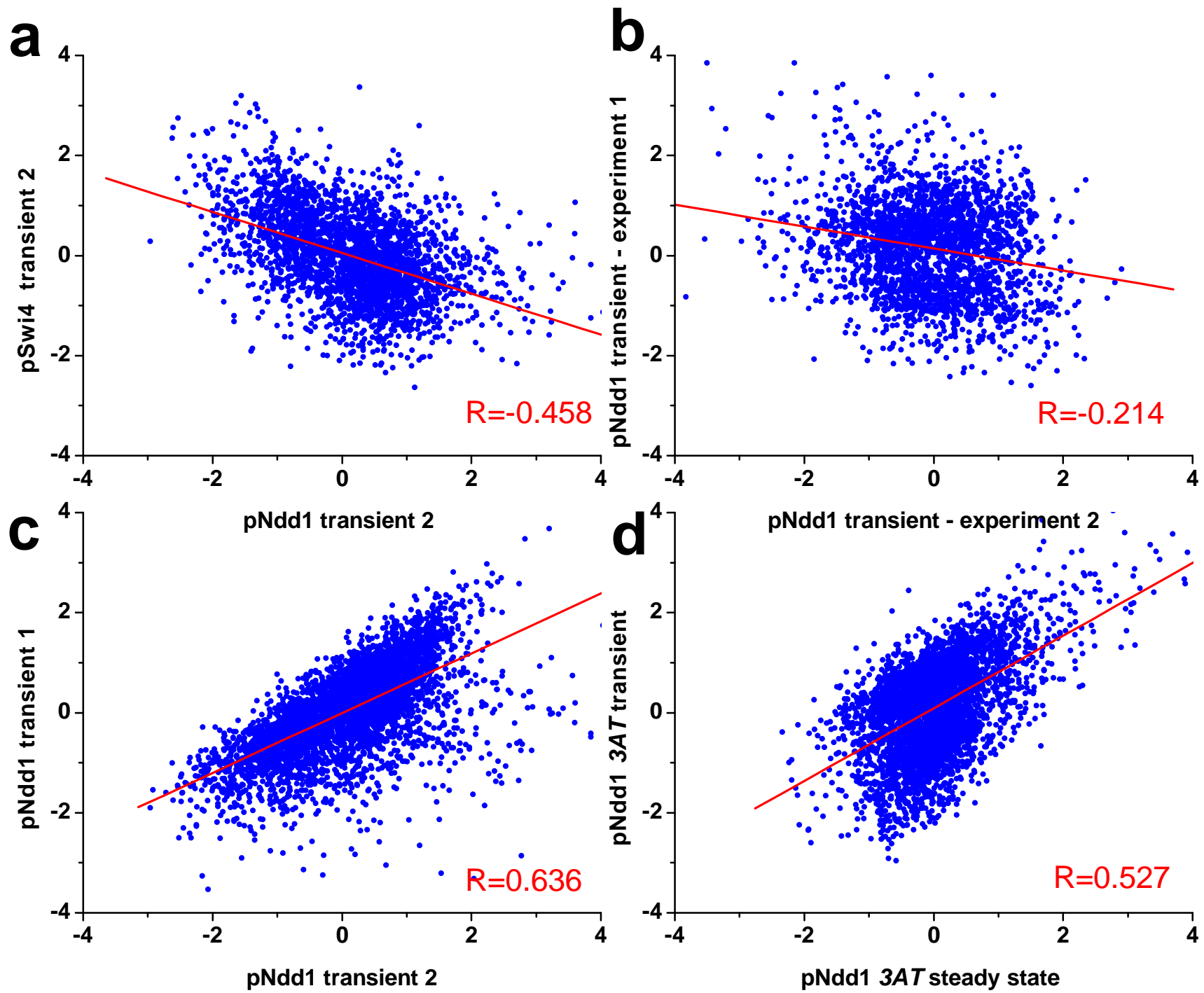

Fig. S9

Supplement: Figure S9 — Comparison between the expression levels in the transient phase for two repeated experiments. The mRNA levels as measured by the DNA arrays (see Methods) at ∼16 hrs after the addition of 3AT to the chemostat medium comparing two populations. (a) Comparison between two transient points of pSwi4-HIS3 and pNdd1-HIS3 strains. (b) Comparison between two transient points of a pair of “twin” pNdd1-HIS3 populations. (c) Comparison between two transient points separated by 7 hrs of the same pNdd1-HIS3 strain population. (d) Comparison between transient and steady state points within the same pNdd1-HIS3 experiment, separated by 390 hrs. The lines are linear fits to the data and the R values are the Pearson correlation coefficients between the experiments on the two axes. Note the negative correlations between two repeated experiments compared to the relatively high positive correlations between two time points within the same experiment. (PDF) [file pone.0045184.s009.pdf]

mRNA levels (A.U.)

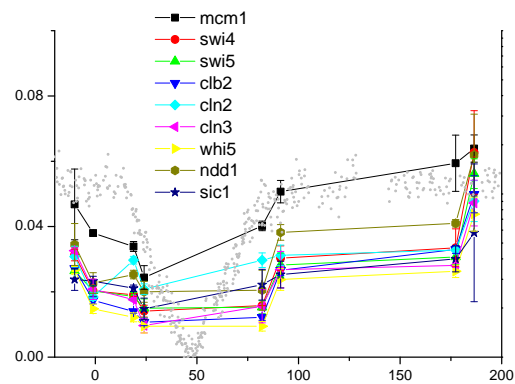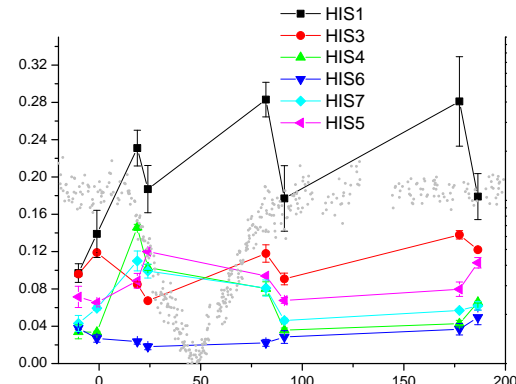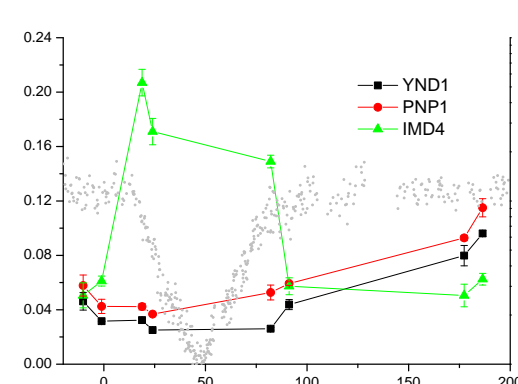

pswi5\_*HIS3*

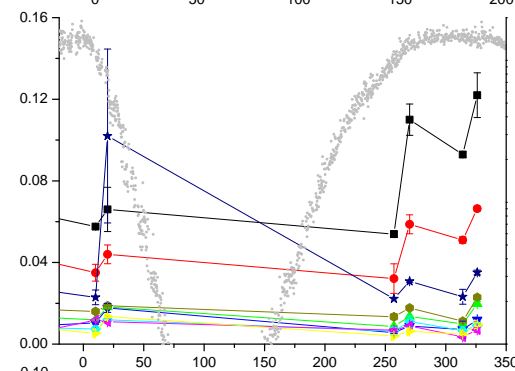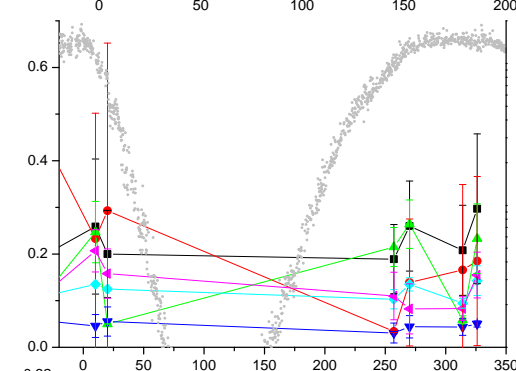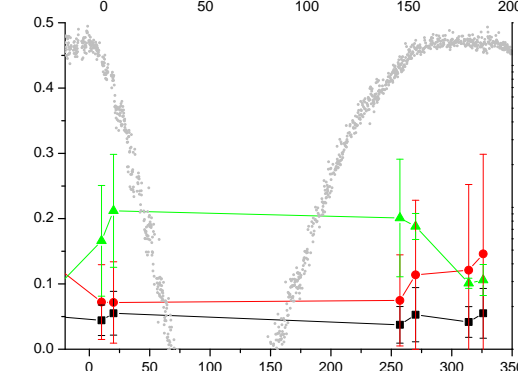

pswi4\_*HIS3*

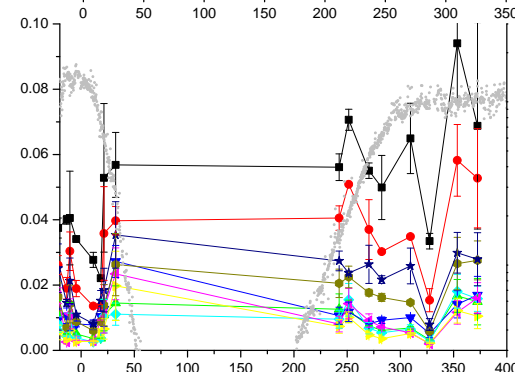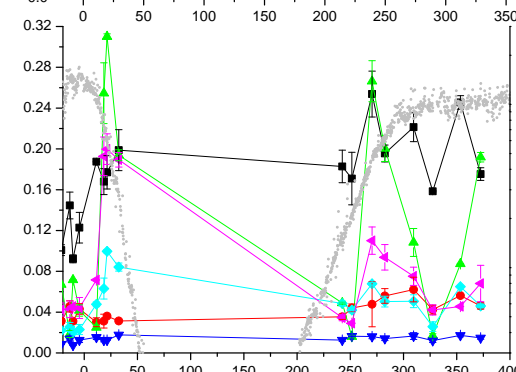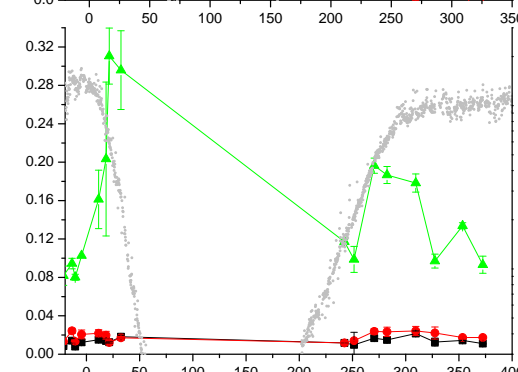

pndd1\_*HIS3*  
*twin a*

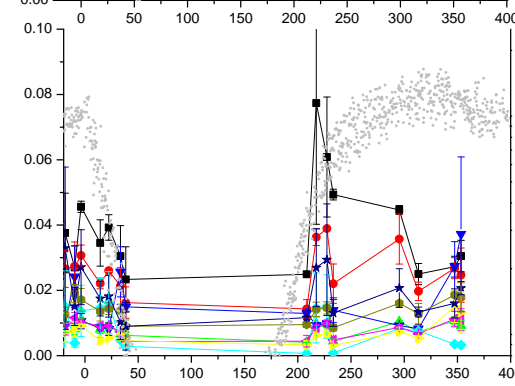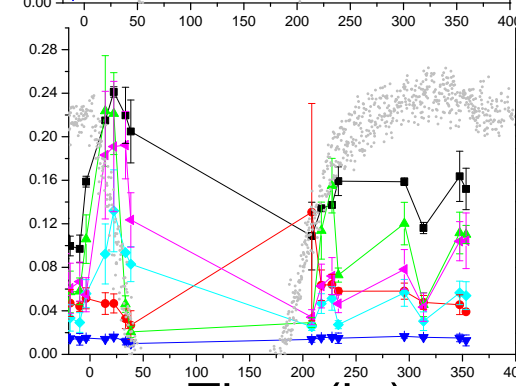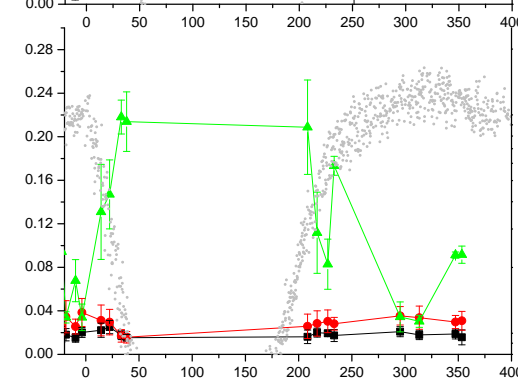

pndd1\_*HIS3*  
*twin b*

Fig. S10

Supplement: Figure S10 — mRNA expression response. Real-time PCR measurements of the mRNA expression levels for genes belonging to: the cell-cycle system (left), histidine pathway (middle), and the purine pathway (right) for cells collected from the same chemostat experiments as in Figure 2 main text. The results are for the following strains: pSwi5-HIS3 (upper row), pSwi4-HIS3 (second row) and pNdd1-His3 for two twin chemostats decoupled just prior to the switch to 3AT (last two rows). The mRNA expression levels are normalized by the expression of ACT1. The results shown are the average over duplicate measurements and the error-bars are the standard deviations. The gray curve in each figure is the OD trace of the corresponding population dynamics. (PDF) [file pone.0045184.s010.pdf]
