# Supplementary material for: Cellular Plasticity Enables Adaptation to Unforeseen Cell-Cycle Rewiring Challenges
Source: PLoS One. 2012 Sep 18;7(9):e45184. doi: 10.1371/journal.pone.0045184 (PMC3445480; doi:10.1371/journal.pone.0045184)
Supplement: Table S1 — GO term groups for a pSwi4- HIS3 population. Gene content of the expression clusters, as well as the number of responding genes, were non-reproducible and varied between nominally identical experiments. There was 8% matching between the genes in the repressed clusters of the pNdd1-HIS3 experiments (the “twin chemostats”), and 9% between the induced clusters. In the pSwi4-HIS3 experiments there were 15% and 10% matching between the genes in the repressed and induced clusters, respectively. Enrichment of biological process was computed for clusters of genes using the GO TermFinder [40], [41]. The annotation search revealed that some of the clusters contained significant GO groups, while other did not. Table S1 depicts the Go term groups in one pSwi4-HIS3 experiment (in the other pSwi4-HIS3 experiment no GO groups were found). (PDF) [file pone.0045184.s011.pdf]

Induced cluster **(616 genes)**			Repressed cluster **(562 genes)**		
**GO term**	**Number of genes**	**P value**	**GO term**	**Number of genes**	**P value**
No significant ontology term could be found			cytoplasmic translation	46 (8.3%)	5.22E-07
			transposition, RNA-mediated	15 (2.7%)	0.00288
