# Supplementary material for: Cellular Plasticity Enables Adaptation to Unforeseen Cell-Cycle Rewiring Challenges
Source: PLoS One. 2012 Sep 18;7(9):e45184. doi: 10.1371/journal.pone.0045184 (PMC3445480; doi:10.1371/journal.pone.0045184)
Supplement: Table S2 — GO term groups, pNdd2 experiment. The same as Table S1 for a pNDD1-HIS3 experiment (in the other pNDD1-HIS3 experiment no GO groups were found). (PDF) [file pone.0045184.s012.pdf]

Induced cluster **(1051 genes)**			Repressed cluster **(835 genes)**		
**GO term**	**Number of genes**	**P value**	**GO term**	**Number of genes**	**P value**
cytoplasmic translation	74 (7.1%)	2.83E-15	No significant ontology term could be found		
translation	112 (10.7%)	3.04E-06			
