# Supplementary material for: Cellular Plasticity Enables Adaptation to Unforeseen Cell-Cycle Rewiring Challenges
Source: PLoS One. 2012 Sep 18;7(9):e45184. doi: 10.1371/journal.pone.0045184 (PMC3445480; doi:10.1371/journal.pone.0045184)
Supplement: Table S3 — Comparison of the genome-wide mRNA expression in our experimentsto genome-wide expressions in stress response and amino acid starvation. (a) The fraction of genes in our experiments that showed at least 2 fold change (induced/repressed) of the expression after the addition of 3AT (4th time point) relative to the initial steady-state and were also induced or repressed in the environmental stress response (ESR, Fig. 3 in [33]). It is clear that the overlap between the expression patterns observed in our experiments and that of the known stress response is insignificant. (b), (c) Amino acid starvation expression data was derived from [42]. The gene expression values that were induced/repressed significantly (at least 2 fold change, p<0.05) in their dataset C (+/−100 mM 3AT) have been compared to our expression data (same as in (a)). The table presents the correlation coefficients between the two experiments. It is clear that there is no correlation between the pattern of expression observed in our experiments and the response to amino acid starvation. (PDF) [file pone.0045184.s013.pdf]

		pSwi4-*HIS3*	pSwi4-*HIS3*	pNdd1-*HIS3*	pNdd1-*HIS3*
a)	Stress response	0.243243<br><br>(135/555)	0.275862<br><br>(152/551)	0.325939<br><br>(191/586)	0.296625<br><br>(167/563)
b)	AA starvation - induced	-0.05042	-0.08681	0.149828	-0.00818
c)	AA starvation -repressed	-0.10444	-0.10241	0.031173	-0.08589
